# Revealing Brownish *Mycena* Diversity in China: New Discoveries and Taxonomic Insights

**DOI:** 10.3390/jof10060439

**Published:** 2024-06-20

**Authors:** Renxiu Wei, Yupeng Ge, Liangliang Qi, Menghui Han, Hui Zeng, Yaping Hu, Li Zou, Xianhao Cheng, Xiaoming Wu, Qin Na

**Affiliations:** 1 Institute of Mycological Science and Technology, School of Agriculture, Ludong University, Yantai 264025, China; wrx_1260@163.com (R.W.); gaiyupeng@126.com (Y.G.); han_mh8128@163.com (M.H.); chengxianhao@sohu.com (X.C.); 2 Institute of Edible Fungi, Fujian Academy of Agricultural Sciences, Fuzhou 350014, China; zenghui@faas.cn; 3 National and Local Joint Engineering Research Center for Breeding & Cultivation of Features Edible Fungi, Fuzhou 350014, China; 4 Microbiology Research Institute, Guangxi Academy of Agricultural Sciences, Nanning 530007, China; qiliangliang529@126.com; 5 Nanjing Institute of Environmental Sciences, Ministry of Ecology and Environment, State Environmental Protection Scientific Observation and Research Station for Ecological Environment of Wuyi Mountains, Nanjing 210042, China; huyap9009@163.com; 6 College of Forestry, Northeast Forestry University, Harbin 150040, China; 13903650896@163.com; 7 Kunyushan National Nature Reserve, Yantai 264112, China; w3215892@163.com

**Keywords:** biodiversity, species complex, new taxon, taxonomy, phylogeny

## Abstract

Within the genus *Mycena*, species exhibiting brownish basidiomata present considerable challenges in identification due to similar coloration. This study underscores the significance of pileipellis types and cheilocystidia characteristics as critical in delimiting brownish *Mycena* species. To clarify the principal taxonomic characters and their utility in distinguishing between brownish *Mycena* species, a morphological taxonomy and phylogenetic analysis were performed. Five new species from China were introduced and characterized through a comprehensive morphological anatomy and phylogenetic substantiation: *M. campanulatihemisphaerica* sp. nov., *M. digitifurcata* sp. nov., *M. kunyuensis* sp. nov., *M. limitis* sp. nov., and *M. oryzifluens* sp. nov. Discussions of these taxa are supplemented with morphological illustrations. The phylogenetic relationships were inferred using Bayesian Inference and Maximum Likelihood methods based on sequences from the internal transcribed spacer and the large subunit regions of nuclear ribosomal RNA. With the addition of these five new species, the worldwide count of brownish *Mycena* increases to 94, and a key to the 29 known species of brownish *Mycena* from China is presented.

## 1. Introduction

*Mycena* (Pers.) Roussel was established in the early 18th century, standing as one of the earliest genera among fungi [1]. Currently, there are over 500 known species, with 2418 listed in the Index Fungorum (https://www.indexfungorum.org, accessed on 27 February 2024), highlighting a remarkable species diversity [2]. *Mycena* species are widely distributed all over the world, and play a crucial role in the decomposition of dead branches, fallen leaves, and rotting wood, which facilitates the material circulation of forest ecosystems [3,4,5,6,7]. Moreover, some species have demonstrated a propensity for enhancing Orchidaceae seed germination, which contributes to the resilience and diversity of ecosystems [8,9,10]. The diverse morphology and color of *Mycena* contribute a high species diversity, while they also present significant taxonomic challenges due to subtle morphological variations and complex microstructures among species [11,12,13,14,15,16]. Taxonomists have proposed several different classification systems, reflecting a varied emphasis on different feature prioritizations [11,12,17,18,19,20]. Presently, the classification system proposed by Maas Geesteranus (1992a, 1992b) has garnered considerable consensus, which divides *Mycena* into 44 sections and considers the color of the pileus and stipe as a primary grouping criterion, for example, sect. *Oregonenses* Maas Geest., sect. *Cyanocephalae* Singer, etc. [11,12].

Brownish *Mycena*, characterized by a yellowish-brown, grayish-brown to dark brown pileus or stipe, predominantly inhabit the northern temperate zone, with fewer occurrences in tropical and subtropical areas [11,12,20,21,22,23,24,25,26,27]. They are categorized into at least nine sections, namely: sect. *Amictae* A.H. Sm. ex Mass Geest., sect. *Basipedes* (Fr.) Quél., sect. *Cinerellae* Singer ex Maas Geest., sect. *Exornatae* Maas Geest., sect. *Filipedes* (Fr.) Quél., sect. *Fragilipedes* (Fr.) Quél., sect. *Hiemalis* Konrad & Maubl., sect. *Mycena* (Pers.) Roussel, and sect. *Rubromarginatae* Singer ex Maas Geest. [17,21,23,26,28,29,30]. Among them, sect. *Mycena* and sect. *Fragilipedes* stand out due to substantial species diversity and typical morphological characteristics. The former is characterized by its light brown, yellowish-brown to sepia pileus, with both the cystidia and pileipellis covered with excrescences. *M. galericulata* (Scop.) Grayis, serving as the type species in genus *Mycena* and as well as in the type sect. *Mycena*, is a prime example of brownish *Mycena*’s characteristic traits. The other section, *Fragilipedes*, is distinguished by its grayish-brown to dark brown pileus, along with smooth cystidia and a pileipellis typically covered with excrescences [11,12,21].

In recent years, the species diversity of *Mycena* has received heightened attention, leading to the publication of many new species, and marking substantial advances in research [13,14,15,26,27,30,31,32]. However, brownish *Mycena* have remained relatively underexplored, with their phylogenetic relationships still unclear and a considerable number of species unknown. Notably, fewer than 30 brownish *Mycena* species have been identified in Asia, with a mere 24 documented in China, compared to the 75 cataloged in Europe and North America [17,20,21,22,23,28,29,30]. The considerable diversity, similar color, and small basidiomata present significant challenges in studying morphological structures, leading to a dominant reason for underestimating the species diversity of brownish *Mycena* in China and across Asia [11,12,13,16,23]. At the same time, the abundant types of cystidia and pileipellis hyphae found in brownish *Mycena* reflect their diverse shapes and suggest a complex evolutionary history. This indicates the species’ adaptability to a wide range of ecological pressures and its ability to thrive in diverse habitats. The diversity of ecological niches and displayed adaptability are concrete manifestations of its complex evolutionary background, reflecting the species’ versatility and flexibility in adapting to environmental changes [21,23].

During our ongoing research and fieldwork aimed at exploring the diversity of *Mycena* in China, five novel species were identified among 14 new specimens through comparative studies with known species. The identification of these novel species was further supported through comprehensive morphological and anatomical research, alongside molecular phylogenetic analyses. This work will expand the variety of brownish *Mycena* in China and provide fundamental information for the systematic study of this group.

## 2. Materials and Methods

### 2.1. Sample Collection and Morphological Description

Specimens used in this study were collected from a broad spectrum of locations across China, spanning Fujian Province, the Guangxi Zhuang Autonomous Region, Heilongjiang Province, Jilin Province, Jiangxi Province, Shandong Province, and Zhejiang Province. During fieldwork, meticulous records were maintained, noting location information, collectors, the collection date, and the ecological habits of each specimen. Simultaneously, the odor and taste of the fresh specimens were recorded. The specimens’ photos were taken using a Canon EOS 90D digital camera (Canon, Tokyo, Japan) equipped with an EF-S60 mm f/2.8 Macro USM (Canon, Tokyo, Japan) and an Olympus E-M1 Mark III camera (Olympus, Tokyo, Japan), coupled with an M. Zuiko Digital Ed 12–40 mm or 60 mm lens (Olympus, Tokyo, Japan). Macro-morphological features, encompassing basidiomata size, color, shape, and surface characteristics were detailed in records based on photographs and field notes. The nomenclature of color descriptions refers to Ridgway (1912) [33]. The extent of pileus marginal striations or sulcations was quantitatively assessed by the ratio of the striation’s or sulcation’s length to the pileus’s radius (n R) [34]. The specimens were dried in an electric oven (Stöckli, A. & J. Stöckli AG, Netstal, Switzerland) at 40 °C and were then placed in a ziplock bag with allochroic silica gel for preservation. All specimens are now preserved in the Fungarium of the Fujian Academy of Agricultural Sciences (FFAAS), China.

Microscopical features were examined with a Lab A1 microscope (Carl Zeiss AG, Jena, Germany) using dried specimens rehydrated in a 5% KOH aqueous solution and stained with a 1% Congo red aqueous solution when necessary. Melzer’s reagent was used to test the amyloidity of basidiospores and lamellar trama [35]. Basidiospores were photographed using ZEN 2.3 (Blue Edition) software (Carl Zeiss Microscopy GmbH, Jena, Germany), with at least 20 basidiospores measured per specimen. For the holotype, a more extensive examination entailed measuring 40 or more basidiospores from each basidiomata. The measured results were presented in the form [*a*/*b*/*c*] (*d*) *e*–*f*–*g* (*h*) × (*i*) *j*–*k*–*l* (*m*) μm [Q = (*n*) *o*–*p* (*q*), Q_m_ = *r* ± *s*]. The abbreviation [*a*/*b*/*c*] represents *a* number of basidiospores measured from *b* number of basidiomata of *c* number of collections; *d* and *h* represent the minimum and maximum lengths (5% extremum), while the range *e*–*g* encompasses the remaining 90% of the measured values, with *f* as the mean length. Width (*i*–*m*) and Q values (*n*–*q*) are analogous representations. If the difference between *d* and *e*, *g* and *h*, *i* and *j*, or *l* and *m* is less than 0.2 μm, and the difference between *n* and *o* or *p* and *q* is less than 0.01 μm, *d*, *h*, *i*, *m*, *n*, or *q* were omitted accordingly. Q represents the “length/width” ratio of a basidiospore. Q_m_ presents the average Q of all basidiospores ± the sample standard deviation [36]. Other microstructures examined include basidia, cheilocystidia, pleurocystidia, pileocystidia, caulocystidia, and the respective compositions of the pileipellis and stipipellis, along with lamellar trama. For each specimen, 20 of these structures were recorded, detailing their sizes, shapes, contents, and other features. The pileipellis samples were specifically cut from the mid-zone between the center and the margin of the pileus, while stipitipellis samples were procured from the middle of the stipe.

The delineation of macro- and microstructures commenced with manual sketches on A4 paper using a pencil and subsequently transposed to tracing paper using a Sakura needle pen (Sakura Color Products Corporation, Osaka, Japan). The sketches were converted to digitalization in TIF format using a Canon LiDE120 scanner (Canon, Tokyo, Japan). Photoshop was then utilized for further refinement and typesetting.

### 2.2. DNA Extraction, PCR Amplification, and Sequencing

DNA extraction from the dried specimens utilized the NuClean Plant Genomic DNA kit (Kangwei Century Biotechnology Co., Beijing, China). PCR amplification followed in a 25 μL reaction mix, comprising 12.5 μL 2× Utaq PCR MasterMix (ZomanBio, Beijing, China), 8.5 μL ddH_2_O, 1 μL each of forward and reverse primers, and 2 μL DNA template [14]. To amplify the nuclear ITS rDNA region (ITS1–5.8S–ITS2), the primer pairs ITS1/ITS4 were employed with the following PCR thermocycling protocol: 94 °C for 4 min, then 34 cycles of 94 °C for 45 s, 52 °C for 45 s, and 72 °C for 1 min, with a final extension of 72 °C for 10 min [37,38]. The large subunit (LSU) of rDNA was amplified using LR0R/LR7 primer pairs with the PCR cycle set to 95 °C for 5 min; 30 cycles of 95 °C for 30 s, 55°C for 30 s, and 72 °C for 1 min; followed by 72 °C for 10 min [16,39]. PCR amplification products underwent agarose gel electrophoresis for validation before the Sanger dideoxy was sequenced by the Beijing Genomics Institute (Beijing, China) [40]. The sequence quality was checked using BioEdit 7.0.9.0, with chromatograms inspected for accuracy [41,42]. Low-quality sequences were enhanced by applying plasmid vectors using VELUTE Gel Mini Purification and pBLUE-T kits (Beijing Zoman Biotechnology Co., Beijing, China) for optimal sequencing results [43]. All newly obtained sequences in this study were submitted to GenBank.

### 2.3. Molecular Phylogeny

The newly obtained sequences were subject to BLAST searches within the NCBI GenBank database (https://www.ncbi.nlm.nih.gov/, accessed on 27 March 2024) to retrieve and download homologous sequences (with nucleotide identities >90%). Furthermore, sequences of species bearing a morphological resemblance to the species in this study were also sourced from GenBank (https://www.ncbi.nlm.nih.gov/genbank/, accessed on 27 March 2024), selecting one or more representatives per species that share a morphological or phylogenetic affinity with the newly described species for preliminary phylogenetic analysis. *M. tenuispinosa* J. Favre and *M. mucoroides* Aronsen were used as outgroup taxa to the root tree. Gaps were treated as missing data after aligning the ITS and LSU datasets independently using the auto strategy (FFT-NS-1, FFT-NS-2, FFT-NS-i or L-INS-i) by MAFFT v.7.110, with gene fragments manually refined and amalgamated in MEGA 5 [44,45,46]. Both Bayesian Inference (BI) and Maximum Likelihood (ML) were utilized for phylogenetic inference. For BI analysis, the optimal nucleotide evolution was estimated using Modeltest 2.3 with the BIC criterion [47]. MrBayes 3.2.6 executed the analysis over 5,000,000 MCMC generations; four chains were conducted with the sampling every 500th generation. The first 25% of trees were discarded as burn-in after the average standard deviation of split frequencies under 0.01, with results aggregated through the “sump” and “sumt” commands [48]. Tracer v.1.7.2 served in the visualization and examination of MCMC trace files from the Bayesian phylogenetic inference [49]. Meanwhile, ML analysis was conducted by RAxMLGUI 2.0 with the GTRGAMMA model and 1000 bootstrap (BS) replicates using default parameters [50]. Finally, the phylogenetic tree was visualized in FigTree v.1.4.3 and refined in Photoshop CS4.

## 3. Results

### 3.1. Phylogenetic Analysis

The molecular analyses dataset consisted of 152 sequences in total, including 28 newly obtained sequences (14 spanning ITS and LSU) and 124 sequences downloaded from GenBank (98 ITS and 26 LSU). Detailed information is provided in Table 1. The aligned dataset culminated in a total of 1743 aligned sites, including gaps (824 ITS, 919 LSU). The average deviation of split frequencies was 0.006, the ESS (effective sample size) was 1908.3, and the average Potential Scale Reduction Factor (PSRF) parameter values = 1.000 after 5,000,000 MCMC generations. The ML phylogenetic analysis yielded a final log-likelihood value of −13,761.724187. The average bootstrap value was 78.5%, indicating a generally high confidence in the tree’s branches. The lowest bootstrap value was 50.0%, suggesting some branches may require further verification, while the highest value was 99.0%, reflecting very high confidence in certain branches. The tree topology effectively depicted the evolutionary relationships among the major taxa, with samples from each novel species clustering together as expected.

**Figure 1:**
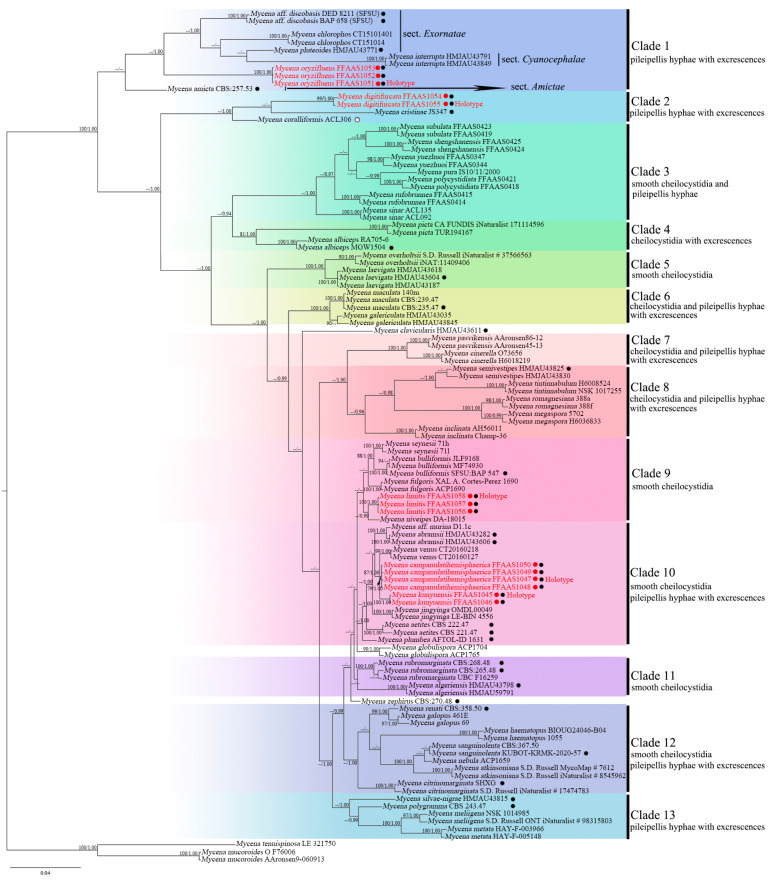
Bayesian Inference tree based on concatenated ITS + LSU dataset. Only branch nodes with both Maximum Likelihood bootstrap support values above 75% and Bayesian posterior probabilities exceeding 0.95 are indicated. Red dots and text represent new taxa, black dots indicate the presence of both ITS and LSU sequences, and white dots signify the presence of only LSU sequences.

The phylogenetic evaluations using ML and BI indicated generally similar topologies for the five newly identified species, although some discrepancies between the methods were observed. Consequently, the BI tree was selected to be shown in Figure 1, incorporating only those nodes supported by bootstrap values of at least 75% and posterior probabilities exceeding 0.95. The phylogenetic structure has resolved into 13 distinct clades, each delineated by shared morphological characteristics of pileipellis hyphae or cheilocystidia among the species within them. While some clades received lower support rates, the topology of the phylogenetic tree still effectively reflects the shared characteristics of groups within these clades, such as smooth or ornamented characteristics.

The five newly identified species demonstrated high support within the phylogenetic tree, with each being robustly placed within clades 1, 2, 9, and 10. These results reinforce the taxonomic standing of these clades and confirm the systematic placement of these new species.

Clade 1 emerges as a monophyletic clade, incorporating *M. oryzifluens* sp. nov. supported by three specimens (FFAAS1051, FFAAS1052, and FFAAS1053). In the BI phylogenetic reconstruction, *M. oryzifluens* sp. nov. clusters closely with other members of Clade 1, indicating a distinct genetic lineage. Although in the ML analysis, *M. oryzifluens* sp. nov. and *M. amicta* (Fr.) Quél. span a broad clade from Clade 2 to Clade 13, their monophyletic status is firmly supported (BS/BPP = 100/1.00). Additionally, species in Clade 1 share the characteristic of non-smooth pileipellis hyphae. Clade 2 incorporates the new species *M. digitifurcata* sp. nov. (FFAAS1054, FFAAS1055), all members sharing the feature of non-smooth pileipellis hyphae. In the BI tree, *M. digitifurcata* sp. nov. is closely related to *M. cristinae*, although this relationship is not as strong in the ML analysis. Despite variations between the BI and ML trees, *M. digitifurcata* sp. nov. is consistently recognized as a monophyletic entity with high statistical support (BS/BPP = 99/1.00). Species of Clade 3 exhibit smooth pileipellis and cheilocystidia, receiving unanimous support (100%) and demonstrating high phylogenetic consistency. Clade 4, although supported at a lower rate (BS/BPP = 81/1.00), presents species with cylindrical excrescences on the cheilocystidia, providing key taxonomic insights. Clades 5 and 6 both exhibit very high support rates (BS/BPP = 100/1.00), where Clade 5 species feature smooth cheilocystidia, while Clade 6 species display excrescences on the cheilocystidia. Clades 7 and 8 also showcase specific morphological characteristics, with Clade 7 having non-smooth pileipellis and Clade 8 featuring non-smooth pileipellis, along with some species having gelatinous layers. Clade 9 includes *M. limitis* sp. nov. (FFAAS1056, FFAAS1057, and FFAAS1058), sharing a close phylogenetic relationship with *M. niveipes* (Murrill) Murrill, along with other species such as *M. seynii* Quél., *M. bulliformis* B.A. Perry & Desjardin, and *M. fulgoris* Cortés-Pérez & Desjardin. Although Clades 9 and 12 form independent groups in BI analysis, Clade 12 nests within Clade 9 in ML analysis. Nonetheless, the position of *M. limitis* sp. nov. remains well-supported (BS/BPP = 100/1.00). Clade 10 comprises the novel species *M. campanulatihemisphaerica* sp. nov. (FFAAS1047, FFAAS1048, and FFAAS1049) and *M. kunyuensis* sp. nov. (FFAAS1045, FFAAS1046), sharing characteristics of smooth cheilocystidia with other species. Clade 11, though not highly supported, includes species that exhibit smooth cheilocystidia. Clade 12 is fully supported (100%), with species displaying smooth cheilocystidia and non-smooth pileipellis, showing clear phylogenetic consistency. Clade 13, similarly supported fully (100%) in the BI tree, shares the characteristic of non-smooth pileipellis among its species.

### 3.2. Taxonomy

**Figure 2:**
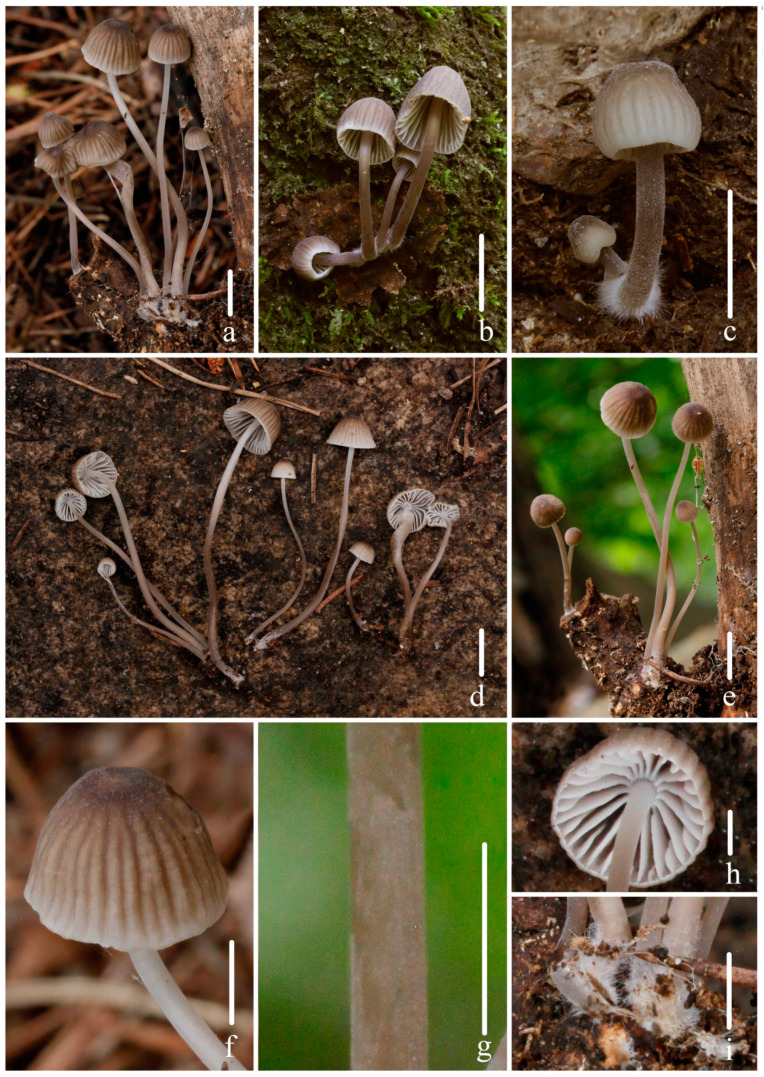
Basidiomata of *Mycena campanulatihemisphaerica* sp. nov. (**a**,**d**–**i**) *FFAAS1047* (holotype). (**b**) *FFAAS1049*; (**c**) *FFAAS1050*; (**f**) Pileus striate-sulcate; (**g**) pubescence on stipe; (**h**) lamellae; (**i**) fibrils length in base of stipe. Bars: (**a**–**e**) = 10 mm; (**f**–**i**) = 5 mm. Photos by Qin Na.

**Figure 3:**
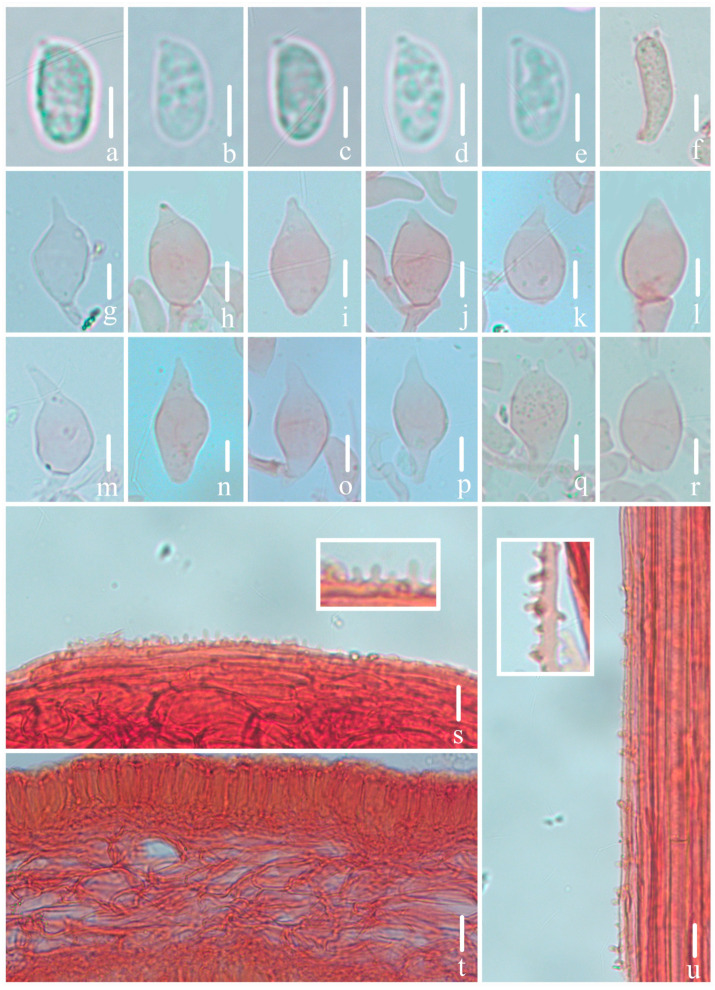
Microscopical features of *Mycena campanulatihemisphaerica* (*FFAAS1047*, holotype). (**a**–**e**) Basidiospores; (**f**) basidium; (**g**–**r**) cheilocystidia; (**s**) pileipellis and upper part of pileipellis hypha with cylindrical excrescences; (**t**) hymenium and lamellar trama; (**u**) stipitipellis and stipitipellis hypha with cylindrical excrescences. Bars: (**a**–**e**) = 5 μm; (**f**–**u**) = 10 μm. Structures (**a**–**e**) were rehydrated in 5% KOH aqueous solution, and (**f**–**u**) were stained in 1% Congo red aqueous solution.

**Figure 4:**
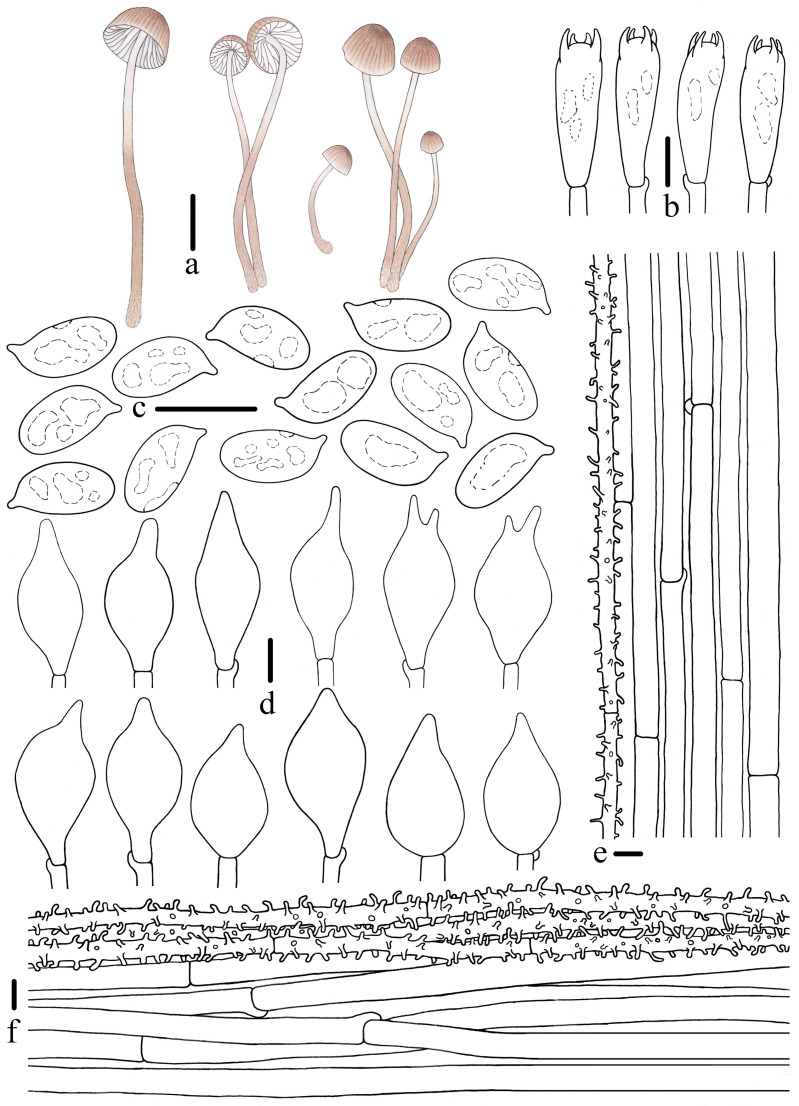
Morphological features of *Mycena campanulatihemisphaerica* (*FFAAS1047*, holotype). (**a**) Basidiomata; (**b**) basidia; (**c**) basidiospores; (**d**) cheilocystidia; (**e**) stipitipellis; (**f**) pileipellis. Bars: (**a**) = 10 mm; (**b**–**f**) = 10 μm. Drawing by Renxiu Wei.

***Mycena campanulatihemisphaerica*** R.X. Wei, X.M. Wu, J. Yu, Y.P. Ge & Q. Na, sp. nov., Figure 2, Figure 3 and Figure 4.

MycoBank no: 853739

Etymology: The epithet *campanulatihemisphaerica* combines the Latin feminine adjectives *campanulata* (bell-shaped) and *hemisphaerica* (half-spherical). This name accurately describes the shape of the pileus, which transitions from bell-shaped to nearly hemispherical.

Holotype: CHINA. Shandong Province, Kunyushan National Nature Reserve, Yantai City, 19 July 2019, leg. Renxiu Wei, Liming Xue, Ruichen Liu, Qin Na, and Yupeng Ge, 516 m asl, *FFAAS1047* (collection no. MY022).

Diagnosis: Pileus fuscous brown, obviously sulcate, basidiospores narrowly ellipsoid to cylindrical, cheilocystidia ovate, ellipsoid, fusiform to sublageniform, some slightly tapered or forked apices, pileipellis and stipitipellis covered with cylindrical excrescences. Differs from *M. venus* by unbranched cheilocystidia and larger basidiospores.

Description: *Pileus* 3–12 mm diam., ovoid, hemispherical to campanulate, sometimes apex with an inconspicuous umbo, margin smooth; *Hair Brown (XLVI17’’’’i) at center, paler towards the margin to Pale Drab-Gray (XLVI17’’’’f), Pale Smoke Gray (XLVI21’’’’f) to White (LIII); pruinose at center, obviously sulcate towards the center up to 0.5–0.8 R, Light Drab (XLVI17’’’’b) to *Hair Brown (XLVI17’’’’i), dry. *Context* White (LIII), fragile, thin. *Lamellae* emarginate to sinuate, 0.2–0.6 mm wide, *L* = 12–16, *l* = 1–2, White (LIII), edge concolorous with face. *Stipe* 19–60 × 0.9–2.0 mm, central, cylindrical, hollow, fragile, *Hair brown (XLVI17’’’’i) at the base, paler toward the apex to White (LIII), finely pubescent, base covered with short, dense, White (LIII) fibrils. *Odor* and *taste* indistinctive.

*Basidiospores* [107/5/4] (8.5)8.8–10.0–12.1 × 4.5–5.3–6.5 μm, [Q =1.74–2.05, Q_m_ = 1.87 ± 0.09] [holotype (40/2/1) 8.8–10.0–12.1 × 4.5–5.3–6.5 μm, Q = 1.74–2.05, Q_m_ = 1.88 ± 0.09], narrowly ellipsoid to cylindrical, hyaline, smooth, thin-walled, amyloid. *Basidia* 21–30 × 7–10 μm, four-spored, rarely two-spored, clavate, hyaline, sterigmata 2–4 μm in length. *Cheilocystidia* 18–36 × 9–16 μm, ovate, ellipsoid, fusiform to sublageniform, apex 1.5–4.2 × 1.2–2.4 μm, tapered, occasionally forked, hyaline, thin-walled. *Pleurocystidia* absent. *Pileipellis* a cutis composed of two layers of hyphae, upper part of hyphae 2.0–5.2 μm diam., parallel, hyaline, thin-walled, covered with cylindrical excrescences, 2.0–7.0 × 1.0–2.4 μm; lower part of hyphae 4.3–6.8 μm diam., interwoven, hyaline, thin-walled, smooth; *pileocystidia* absent. *Lamellae trama* subcellular, composed of subglobose, ellipsoid, and narrowly ellipsoid cells, 23–43 × 17–25 μm, hyaline, dextrinoid. *Stipitipellis* a cutis composed of a hypha, 1.9–4.8 μm diam., hyaline, thin-walled, covered with cylindrical excrescences, 1.7–4.1 × 0.6–1.6 μm; *caulocystidia* absent. *Clamps* present in all tissues, but rarely observed in context.

Habit and habitat: Fascicled or scattered on rotten branches in mixed broadleaf–conifer forests, mainly under trees of *Picea*, *Pinus*, and *Quercus*.

Known distribution: Fujian Province, Jiangxi Province, Shandong Province, China.

Additional material examined: China. Fujian Province, Junzifeng National Nature Conservation Area, Mingxi County, Sanming City, 2 May 2021, leg. Renxiu Wei, Binrong Ke, Hui Zeng, Junqing Yan, Qin Na, and Yupeng Ge, 824 m asl, *FFAAS1049* (collection no. MY0267); Jiangxi Province, Jiangxi Agricultural University, Nanchang City, 22 April 2021, leg. Renxiu Wei, Junqing Yan, and Qin Na, 59 m asl, *FFAAS1048* (collection no. MY056); Shandong Province, Mengshan Scenic Area, Mengyin County, Linyi City, 20 July 2021, leg. Renxiu Wei, Yulan Sun, Zewei Liu, Qin Na, and Yupeng Ge, 962 m asl, *FFAAS1050* (collection no. MY0389).

Notes: *M. venus* exhibits morphological affinities with *M. campanulatihemisphaerica*, particularly in the characteristics of the pileus and stipe. However, *M. venus* is distinguished by its branched cheilocystidia and notably smaller basidiospores, measuring (4.8) 6.8–7.7 (8.7) × (3.2) 3.8–4.8 (5.4) μm, with a Q_m_ of 1.69 [30]. *M. silvae-nigrae* Maas Geest. & Schwöbel and *M. leptocephala* (Pers.) Gillet share similarities with *M. campanulatihemisphaerica* in pileus color. Nonetheless, *M. silvae-nigrae* is set apart by its uniformly dark brown stipe and the presence of pleurocystidia [21,23,28]; *M. leptocephala* is differentiated by presenting caulocystidia and pleurocystidia [20,21,23,28,29]. Microscopically, *M. abramsii* (Murrill) Murrill and *M. scirpicola* M. Villarreal, Heykoop, Esteve-Rav. & Maas Geest. also display cheilocystidia of a similar morphology, such as clavate, fusiform, and sublageniform to cylindrical, but *M. campanulatihemisphaerica* is distinguishable from these species by its fuscous brown pileus and the absence of both pleurocystidia and caulocystidia [20,21,23,28,29,67,68]. Additionally, some cheilocystidia were observed with characteristics such as slightly tapered or forked apices, resembling subglobose and subclavate cells, suggesting they are in an early phase of cheilocystidia.

**Figure 5:**
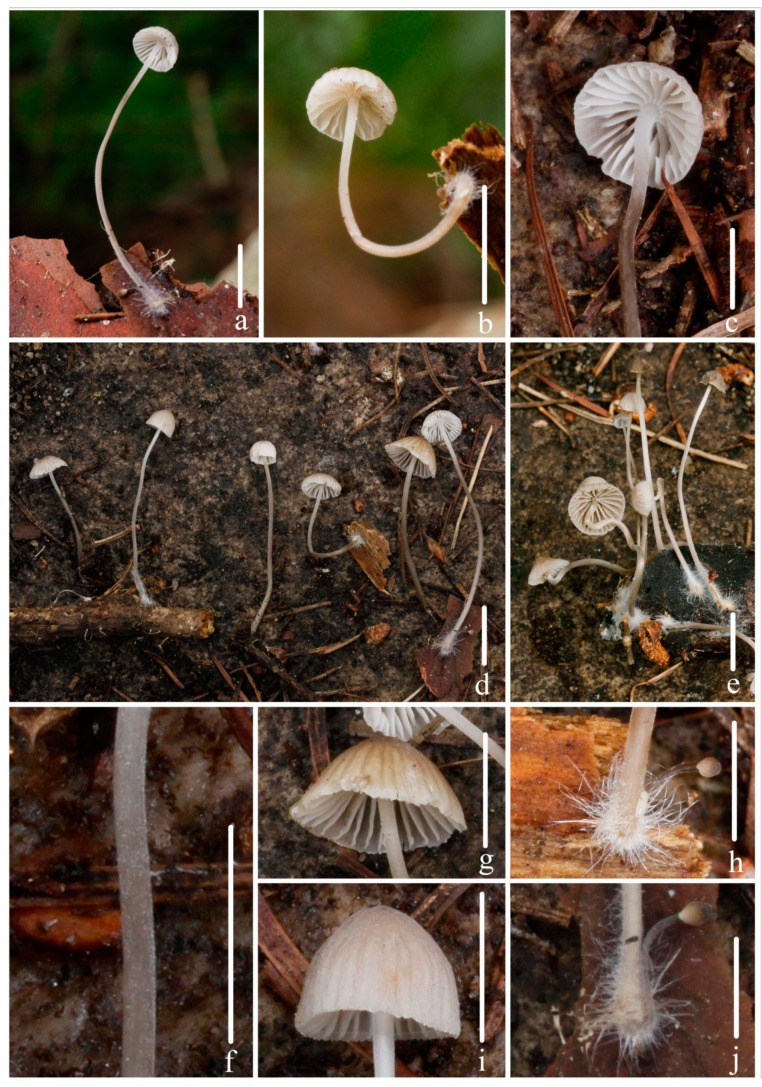
Basidiomata of *Mycena kunyuensis* sp. nov. (**a**–**d**,**f**–**j**) *FFAAS1045*; (**e**) *FFAAS1046* (holotype); (**f**) The surface of the stipe is pubescent; (**g**,**i**) Pileus color and striate-sulcate; (**h**,**j**) Fibrils length in the base of the stipe. Bars: (**a**–**d**) = 10 mm; (**e**–**j**) = 5 mm. Photos by Qin Na.

**Figure 6:**
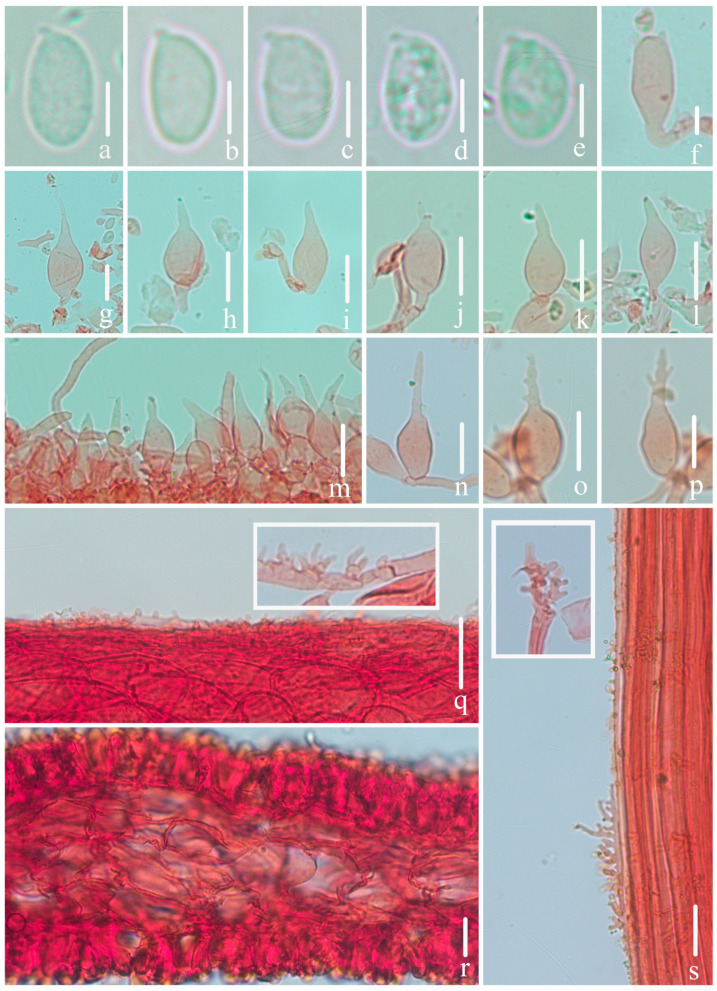
Microscopical features of *Mycena kunyuensis* (*FFAAS1045*, holotype). (**a**–**e**) Basidiospores; (**f**) basidium; (**g**–**p**) cheilocystidia; (**q**) pileipellis and pileipellis hypha with cylindrical excrescences; (**r**) Hymenia and lamellar trama; (**s**) stipitipellis and stipitipellis hypha with cylindrical excrescences. Bars: (**a**–**f**) = 5 μm; (**g**–**s**) = 20 μm. Structures (**a**–**e**) were rehydrated in 5% KOH aqueous solution, and (**f**–**s**) were stained in 1% Congo red aqueous solution.

**Figure 7:**
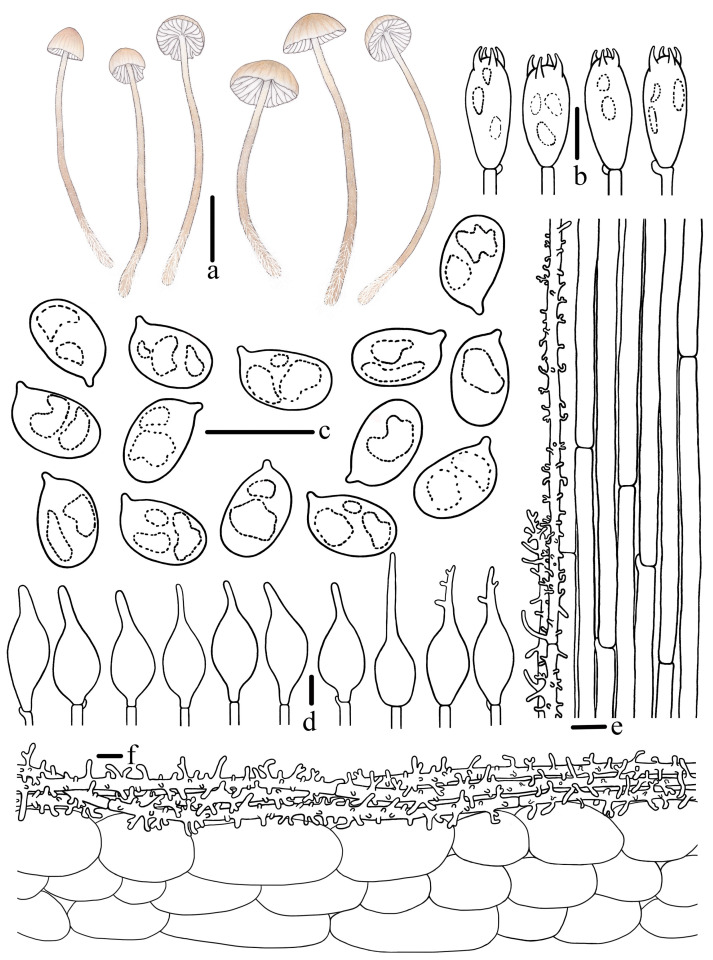
Morphological features of *Mycena kunyuensis* (*FFAAS1045*, holotype). (**a**) Basidiomata; (**b**) basidia; (**c**) basidiospores; (**d**) cheilocystidia; (**e**) stipitipellis; (**f**) pileipellis and context. Bars: (**a**) = 10 mm; (**b**–**f**) = 10 μm. Drawing by Renxiu Wei.

***Mycena kunyuensis*** R.X. Wei, X.M. Wu, M.Z. Chen, Y.P. Ge & Q. Na, sp. nov., Figure 5, Figure 6 and Figure 7.

MycoBank no: 853782

Etymology: The epithet *kunyuensis* refers to the Kunyu mountain where the holotype was discovered.

Holotype: CHINA. Shandong Province, Kunyushan National Nature Reserve, Yantai City, 19 July 2019, leg. Renxiu Wei, Liming Xue, Ruichen Liu, Qin Na, and Yupeng Ge, 513 m asl, *FFAAS1045* (collection no. MY021).

Diagnosis: Pileus pale olive-buff to olive-buff, basidiospores ellipsoid to narrowly ellipsoid, cheilocystidia lageniform, obpyriform, obclavate to fusiform, with a narrow neck, apex tapered, pileipellis and stipitipellis covered with cylindrical excrescences. Differs from *M. abramsii* by smaller basidiomata and absent pleurocystidia.

Description: *Pileus* 5–11 mm diam., fusiform to campanulate when young, slightly campanulate, conical, hemispherical to plano-convex with age, margin slightly serrulate, occasionally cracked when old; Citrine-Drab (XL21’’’i), Deep Quaker Drab (LI1’’’’’i) at center, paler towards margin to *Olive-Buff (XL21’’’d) when young, Citrine-Drab (XL21’’’i), Pale Mouse Gray (LI15’’’’’d) at center, gradually paler towards margin to Pale Olive-Buff (XL21’’’f), Pallid Mouse Gray (LI15’’’’’f) to White (LIII) when matured; initially pruinose, when matured glabrescent, undistinguished to clear striate towards the center up to 0.5–0.6 R, dry. *Context* White (LIII), fragile, thin. *Lamellae* sinuate, 0.3–0.6 mm wide, *L* = 14–18, *l* = 1, White (LIII), edge concolorous. *Stipe* 33–46 × 0.8–1.1 mm, central, clavate to cylindrical, hollow, fragile, in young stages; Deep Quaker Drab (LI1’’’’’i), *Hair Brown (XLVI17’’’’i), when matured White (LIII) in the upper part, Pale Smoke Gray (XLVI21’’’’f), Light Grayish Olive (XLVI21’’’’b) towards the base, pruinose when young, then apex to middle part finely pubescent, almost glabrous in the lower part, base slightly swollen, densely covered with long White (LIII) fibrils. *Odor* and *taste* indistinctive.

*Basidiospores* [65/3/2] (7.0) 7.4–9.3–11.5 (11.8) × 4.6–5.8–6.9 μm, [Q = (1.40) 1.43–1.77 (1.79), Q_m_ = 1.60 ± 0.09] [holotype (40/2/1) (7.0) 7.4–8.8–10.4 × 4.6–5.6–6.8 μm, Q = (1.40) 1.43–1.77, Q_m_ = 1.56 ± 0.08], ellipsoid to narrowly ellipsoid, hyaline, smooth (under oil), thin-walled, amyloid. *Basidia* 16–23 × 5–9 μm, four-spored, rarely two-spored, clavate, hyaline, sterigmata 2.1–4.2 μm in length. *Cheilocystidia* 28–59 × 8–20 μm, lageniform, obpyriform, obclavate to fusiform, with a narrow neck, apex tapered, smooth, sometimes neck with several excrescences, and occasionally base constricted to cylindrical, hyaline, thin-walled. *Pleurocystidia* absent. *Pileipellis* a cutis composed of parallel hyphae, 2.5–5.1 μm diam., thin-walled, densely covered with cylindrical excrescences, 1.6–9.2 × 1.0–2.6 μm, occasionally forked; *pileocystidia* absent. *Lamellae trama* cellular, composed of subglobose, ellipsoid, and narrowly ellipsoid cells, 24–48 × 21–25 μm, hyaline, dextrinoid. *Stipitipellis* a cutis composed of a hypha, (1.7) 2.1–4.6 μm diam., covered with cylindrical excrescences, 0.6–10.2 (15.3) × 0.9–2.9 μm, occasionally forked, hyaline, thin-walled; *caulocystidia* absent. *Clamps* present in all tissues, but rarely observed in context.

Habit and habitat: Scattered on rotten branches in mixed broadleaf–conifer forests, mainly under trees of *Picea*, *Pinus*, and *Quercus*.

Known distribution: Shandong Province, China.

Additional material examined: CHINA. Shandong Province, Kunyushan National Nature Reserve, Yantai City, 19 July 2019, leg. Renxiu Wei, Liming Xue, Ruichen Liu, Qin Na, and Yupeng Ge, 524 m asl, *FFAAS1046* (collection no. MY023).

**Figure 8:**
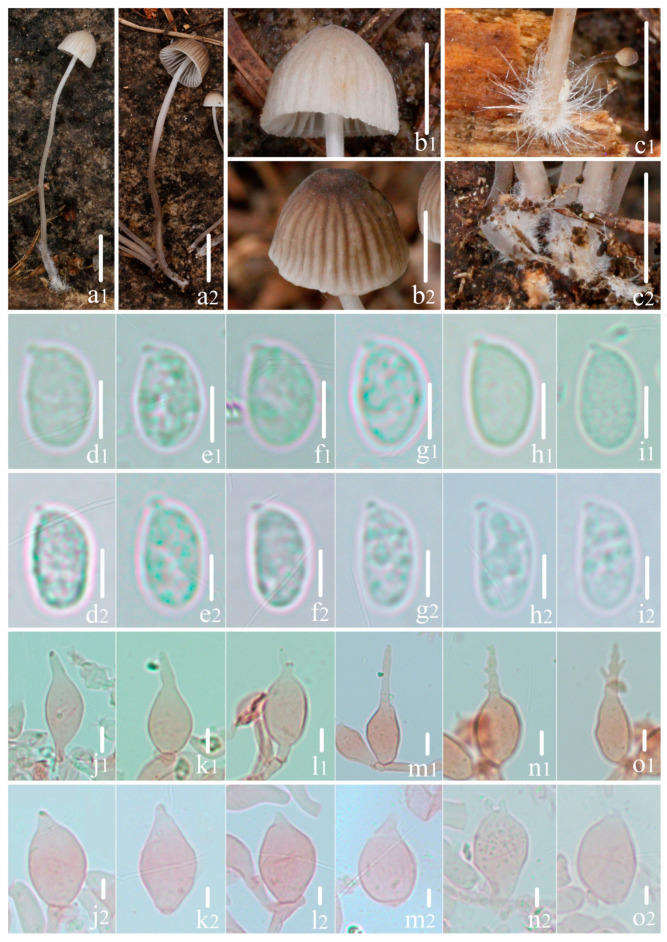
Morphological features of *Mycena kunyuensis* and *Mycena campanulatihemisphaerica*. (**1**) *Mycena kunyuensis*; (**2**) *Mycena campanulatihemisphaerica*. (**a**) Basidiomata color; (**b**) pileus striate-sulcate; (**c**) fibrils length in base of stipe; (**d**–**i**) basidiospores shape; (**j**–**o**) cheilocystidia shape. Bars: (**a**) = 10 mm; (**b**,**c**) = 5 mm; (**d**–**o**) = 5 μm.

Notes: *M. abramsii* is the closest species to *M. kunyuensis*, sharing numerous macroscopic and microscopic characters. Nevertheless, *M. abramsii* is distinguished by its much larger basidiomata and the presence of pleurocystidia [20,21,23,28,29,67,68]. In addition, taxa such as *M. fagetorum* (Fr.) Gillet, *M. metata* (Fr.) P. Kumm. and *M. filopes* (Bull.) P. Kumm. exhibit a similar pileus color, but distinct differences are noted: *M. fagetorum* is distinguished by its clavate cheilocystidia, which are prominently covered by coarse excrescences [28]; *M. metata* is differentiated by its clavate and obovoid cheilocystidia, and presenting pleurocystidia [21,23,28,29]; and *M. filopes* is recognized by its excrescence-covered cheilocystidia and the presence of caulocystidia [21,23,28,29]. *M. aetites* (Fr.) Quél. also presents lageniform cheilocystidia, but it is uniquely identified by its pileus, which ranges from black to dark brown, and its gelatinized pileipellis [23,28]. Comparatively, *M. kunyuensis* and *M. campanulatihemisphaerica* exhibit similarities in some macroscopic and microscopic characters. However, *M. campanulatihemisphaerica* can be distinguished by its shorter fibrils-covered stipe; fuscous brown, markedly sulcate pileus, with its margin being slightly serrulate; larger basidiospores; and cheilocystidia that fork at the apex (Figure 8).

**Figure 9:**
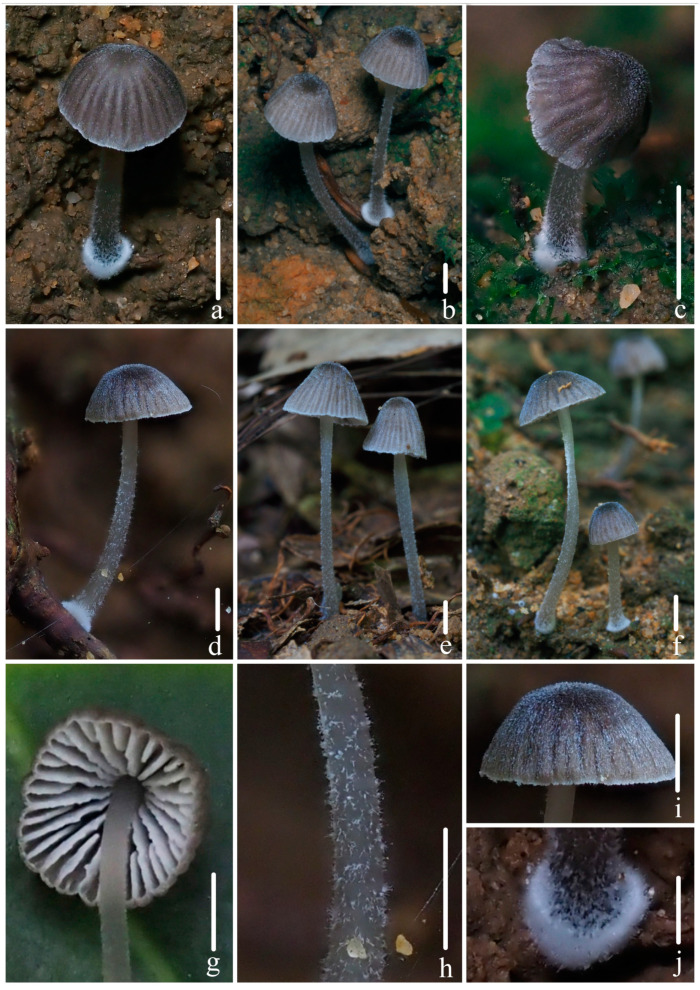
Basidiomata of *Mycena oryzifluens* sp. nov. (**a**,**c**,**j**) *FFAAS1051* (holotype); (**g**) *FFAAS1052*; (**b**,**d**–**f**,**h**,**i**) *FFAAS1053*; (**g**) Lamellae; (**h**) The surface of the stipe is pruinose and pubescent; (**i**) The surface of the pileus is pruinose and pubescent; (**j**) Tomentum length in the base of the stipe. Bars: (**a**–**i**) = 2 mm; (**j**) = 1 mm. Photos by Yupeng Ge.

**Figure 10:**
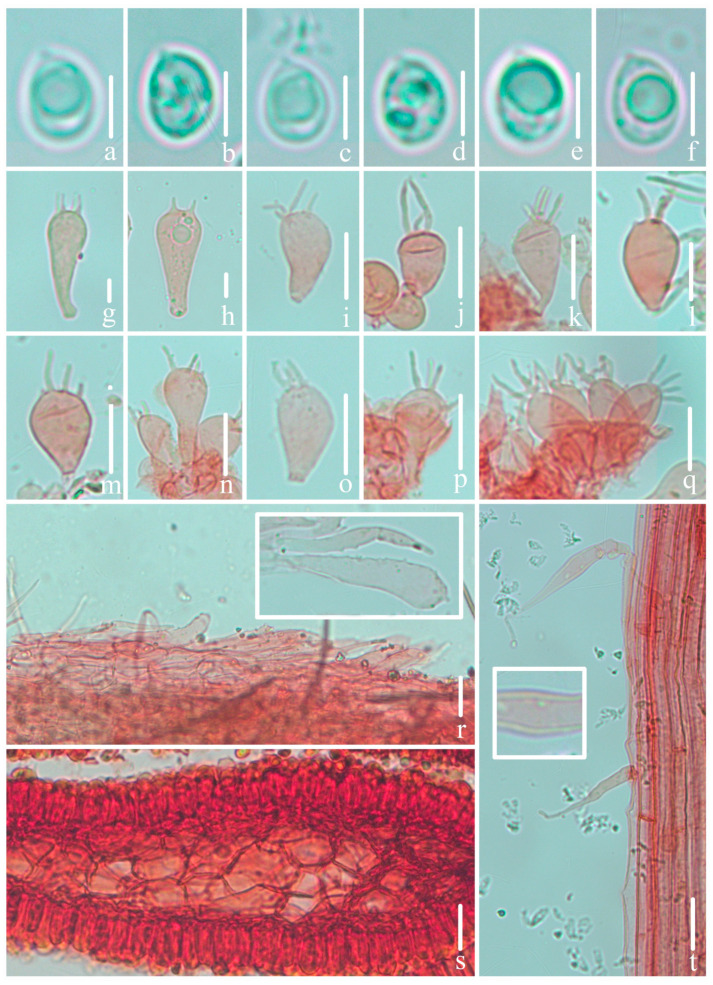
Microscopical features of *Mycena oryzifluens* (*FFAAS1051*, holotype). (**a**–**f**) Basidiospores; (**g**,**h**) basidia; (**i**–**q**) cheilocystidia; (**r**) pileipellis and terminal cells; (**s**) hymenia and lamellar trama; (**t**) stipitipellis and thick-walled caulocystidia. Bars: (**a**–**h**) = 5 μm; (**i**–**t**) = 20 μm. Structures (**a**–**f**) were rehydrated in 5% KOH aqueous solution, and (**g**–**t**) were stained in 1% Congo red aqueous solution.

**Figure 11:**
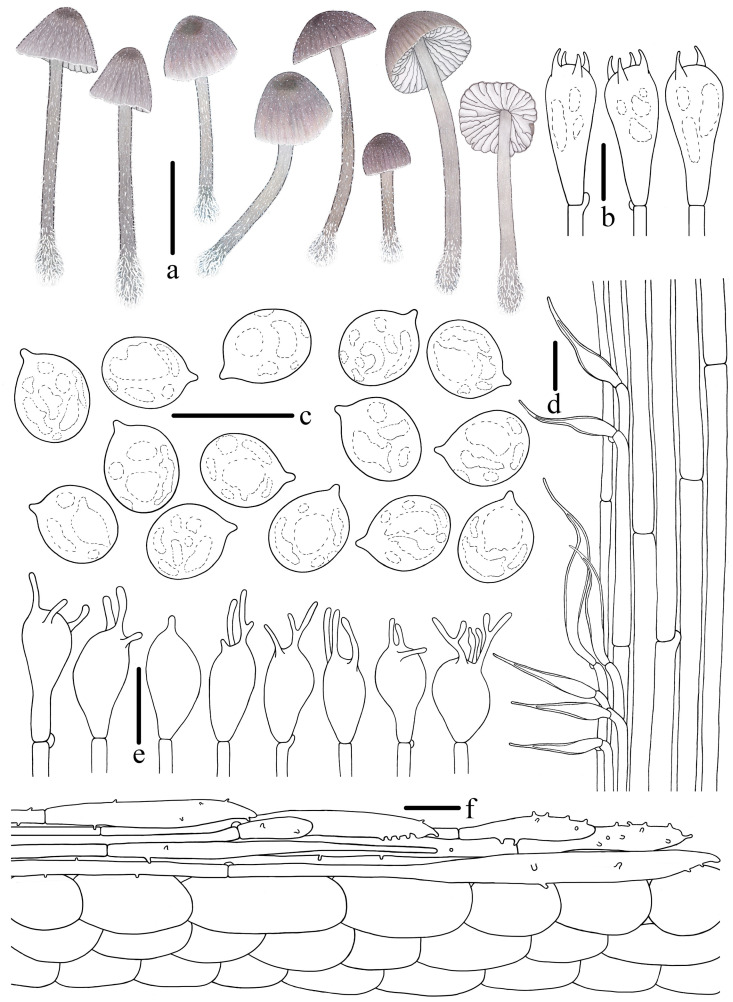
Morphological features of *Mycena oryzifluens* (*FFAAS1051*, holotype). (**a**) Basidiomata; (**b**) basidia; (**c**) basidiospores; (**d**) stipitipellis; (**e**) cheilocystidia; (**f**) pileipellis and context. Bars: (**a**) = 5 mm; (**b**,**c**) = 10 μm; (**d**–**f**) = 20 μm. Drawing by Renxiu Wei.

***Mycena oryzifluens*** R.X. Wei, L.L. Qi, Y.P. Ge & Q. Na, sp. nov., Figure 9, Figure 10 and Figure 11.

MycoBank no: 853783

Etymology: The epithet *oryzifluens* is derived from the Latin word *oryzi*, meaning rice, and fluens, meaning flowing. This name reflects the rice-like appearance of the white, pruinose, and pubescent stipe and connects to the folklore of the type locality, where a legend speaks of a flowing rice cave, symbolizing abundance and continuity.

Holotype: CHINA. Guangxi Zhuang Autonomous Region, Qingxiushan Scenic Spot, Nanning City, 14 July 2022, leg. Renxiu Wei, LiangLiang Qi, Liying Li, Yongqiang Hu, and Yupeng Ge, 164 m asl, *FFAAS1051* (collection no. MY0870).

Diagnosis: Pileus plumbeous to gray, finely white pruinose and pubescent, basidiospores subglobose to broadly ellipsoid, cheilocystidia clavate, ovoid, obpyriform, with finger-like branches in the apex, pileipellis with non-smooth terminal cells, caulocystidia thick-walled. Different from *M. cretata* Aronsen by clavate cheilocystidia with cylindrical branches, thick-walled caulocystidia, and absent pleurocystidia.

Description: *Pileus* 2.1–6.0 mm diam., campanulate when young, then hemispherical to oblate hemispherical; Dark Plumbeous (LII49’’’’’i) at center, paler towards margin to Plumbeous (LII49’’’’’b) when young, paler towards margin to French Gray (LII49’’’’’f), *Lilac Gray (LIII59’’’’’f) to White (LIII) with age; finely White (LIII) pruinose and pubescent, especially dense White (LIII) pubescence surrounds the margin, sulcate towards the center up to 0.5–0.6 R, Dark Vinaceous Gray (LII59’’’’’k), Vinaceous Gray(L69’’’’’d) when young, Light Violet Gray(LII59’’’’’b) to Deep Violet Gray (LII59’’’’’i) when matured, dry. *Context* White (LIII), fragile, thin. *Lamellae* adnate, 0.3–0.5 mm wide, *L* = 16–25, *l* = 1–2, White (LIII), edge serrulate, concolorous with face. *Stipe* 5–20 × 0.3–1.0 mm, center, cylindrical, hollow, fragile, Deep Purplish Gray (LIII67’’’’i) when young, then paler to Light Violet-Gray (LII59’’’’’b), Hathi Gray (LII35’’’’’b) with age, finely White (LIII) pruinose and pubescent, base densely covered with White (LIII) tomentum. *Odor* and *taste* indistinctive.

*Basidiospores* [96/4/3] 6.0–6.6–7.5 (7.7) × 4.6–5.3–6.3 μm, [Q = 1.10–1.45, Q_m_ = 1.24 ± 0.07] [holotype (40/2/1) 6.2–6.8–7.4 (7.7) × (4.8)5.0–5.6–6.3 μm, Q = 1.10–1.37, Q_m_ = 1.20 ± 0.05], subglobose to broadly ellipsoid, hyaline, smooth (under oil), thin-walled, amyloid. *Basidia* 17–27 × 7–10 μm, two- and four-spored, clavate, hyaline, sterigmata 1.7–4.7 μm in length. *Cheilocystidia* 15–36 × 8–18 μm, clavate, ovoid, obpyriform, with one to several finger-like branches at apex, 1.0–17.1 × 0.6–2.6 μm, sometimes branches with furcate excrescences, hyaline, thin-walled. *Pleurocystidia* absent. *Pileipellis* a cutis composed of parallel hyphae, 2.7–5.2 μm diam., hyaline, thin-walled, terminal cells often swollen, 34–103 × 5–15 μm, clavate to cylindrical, with sparse nodulose excrescences, 0.5–3.0 × 0.5–1.0 μm; *pileocystidia* absent. *Lamellae trama* cellular, composed of subglobose, ellipsoid and narrowly ellipsoid cells, 20–50 × 15–30 μm, hyaline, dextrinoid. *Stipitipellis* a cutis composed of a hypha, 2.8–5.7 μm diam., hyaline, thin-walled; *caulocystidia* 44–107 × 4–10 μm, cylindrical, with a narrow protuberance in the apex, thick-walled (0.6–1.7 μm thick), smooth, hyaline. *Clamps* present in all tissues, but rarely observed in context.

Habit and habitat: Scattered on litter layers and rotten branches in broad-leaved mixed forests, mainly under trees of *Ficus* and *Parashorea*.

Known distribution: Guangxi Zhuang Autonomous Region, China.

Additional material examined: China. Guangxi Zhuang Autonomous Region, Qingxiushan Scenic Spot, Nanning City, 14 July 2022, leg. Renxiu Wei, LiangLiang Qi, Liying Li, Yongqiang Hu, and Yupeng Ge, 185 m asl, *FFAAS1052* (collection no. MY0871), 191 m asl, *FFAAS1053* (collection no. MY0872).

Notes: Macroscopically, *M. cretata* shares a similar pileus color with *M. oryzifluens* but is distinguished by the presence of pleurocystidia and thin-walled caulocystidia, with its cheilocystidia lacking finger-like extensions [28]. Microscopically, both *M. tallangattensis* Grgur and *M. scirpicola* feature thick-walled caulocystidia. However, *M. tallangattensis* is identified by its thick-walled cheilocystidia and the presence of pleurocystidia [17], while *M. scirpicola* is noted for its elongated basidiospores and the absence of branched cheilocystidia [23,28]. Additionally, taxa *M. tristis* Maas Geest., *M. clavularis* (Batsch) Sacc., *M. tenuispinosa*, and *M. mucor* (Batsch) Quél. exhibit cheilocystidia of a similar morphology, but *M. oryzifluens* is distinctively characterized by its plumbeous to gray pileus, basidiospores that are subglobose to broadly ellipsoid, and thick-walled caulocystidia [28].

**Figure 12:**
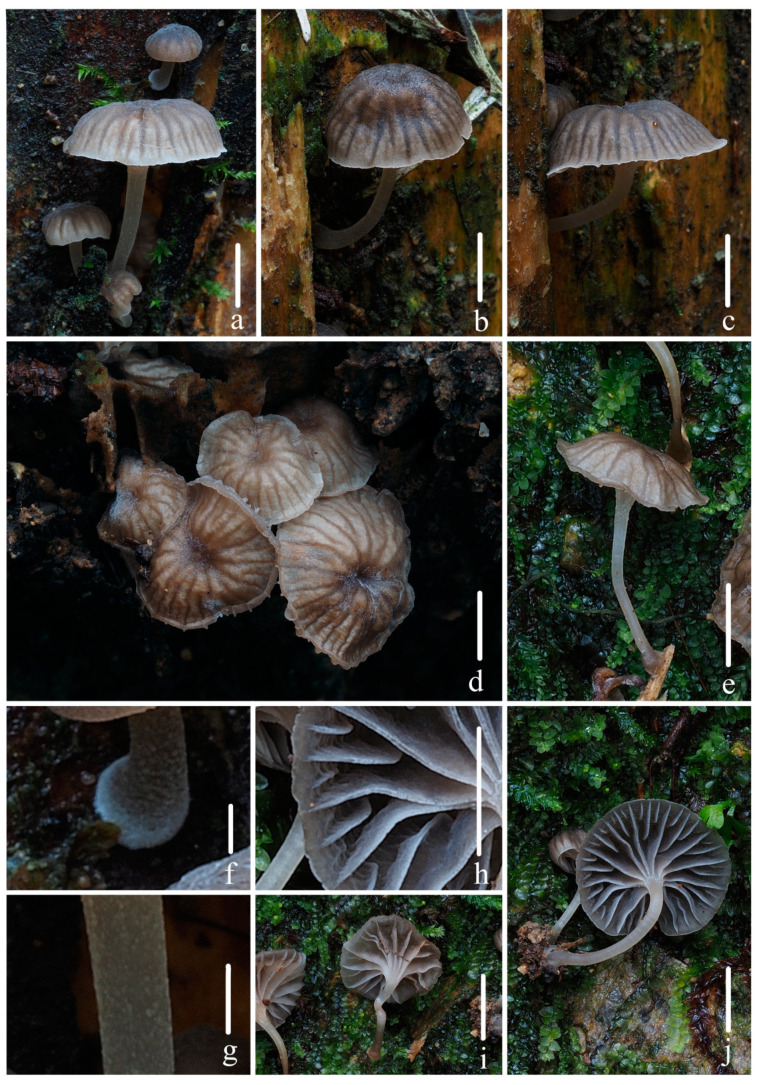
Basidiomata of *Mycena digitifurcata* sp. nov. (**a**–**j**) *FFAAS1055* (holotype); (**f**) Tomentum in the base of the stipe; (**g**) The surface of the stipe is pruinose and pubescent; (**h**–**j**) Lamellae. Bars: (**a**–**j**) = 5 mm. Photos by Qin Na.

**Figure 13:**
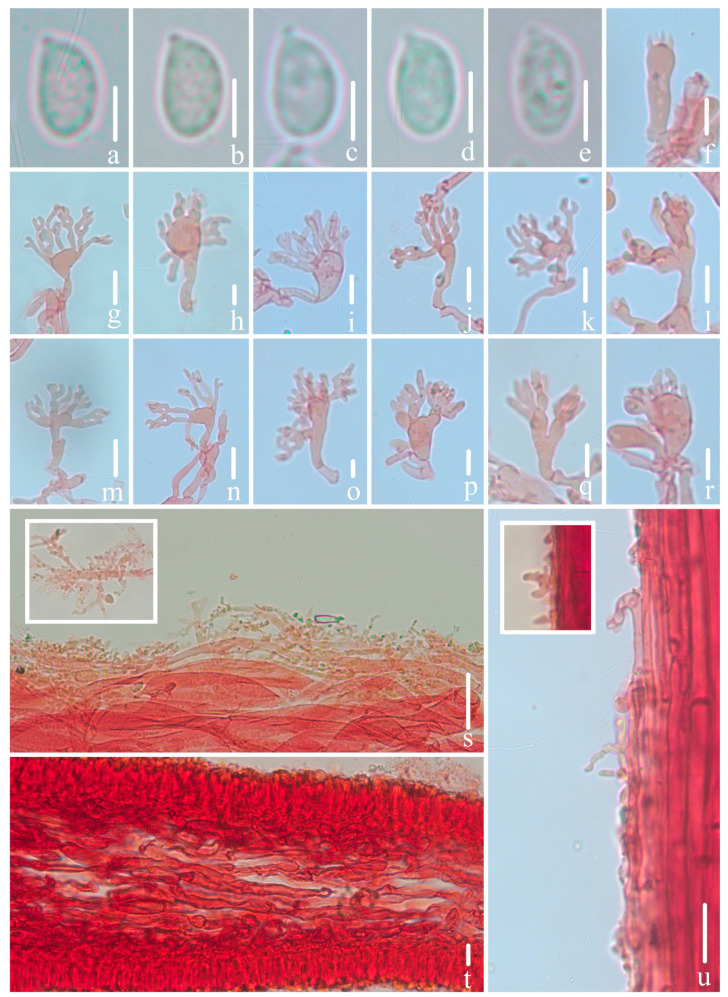
Microscopical features of *Mycena digitifurcata (FFAAS1055*, holotype). (**a**–**e**) Basidiospores; (**f**) basidium; (**g**–**r**) cheilocystidia; (**s**) pileipellis and pileipellis hypha with cylindrical excrescences; (**t**) hymenia and lamellar trama; (**u**) stipitipellis and stipitipellis hypha with cylindrical excrescences. Bars: (**a**–**e**) = 5 μm; (**f**–**r**) = 10 μm; (**s**–**u**) = 20 μm. Structures (**a**–**e**) were rehydrated in 5% KOH aqueous solution, and (**f**–**u**) were stained in 1% Congo red aqueous solution.

**Figure 14:**
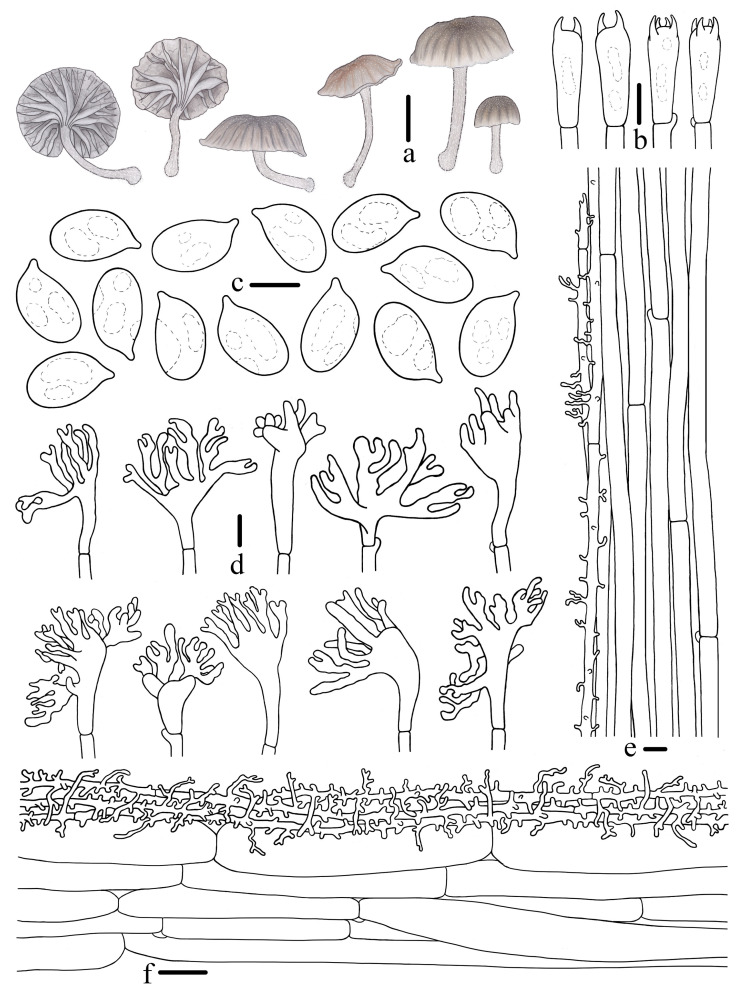
Morphological features of *Mycena digitifurcata* (*FFAAS1055*, holotype). (**a**) Basidiomata; (**b**) basidia; (**c**) basidiospores; (**d**) cheilocystidia; (**e**) stipitipellis; (**f**) pileipellis and context. Bars: (**a**) = 5 mm; (**b**,**d**–**f**) = 10 μm; (**c**) = 5 μm. Drawing by Renxiu Wei.

***Mycena digitifurcata*** R.X. Wei, H. Zeng, Y.P. Ge & Q. Na, sp. nov., Figure 12, Figure 13 and Figure 14.

MycoBank no: 853784

Etymology: The epithet *digitifurcata* derives from the Latin words ‘*digitus*’, meaning ‘finger’, and ‘furcatus’, meaning ‘forked’. This name is chosen to describe the distinctive finger-like and forked projections at the apex of the cheilocystidia.

Holotype: CHINA. Zhejiang Province, Baiyun National Forest Park, Lishui City, 2 August 2021, leg. Renxiu Wei, Zewei Liu, Qin Na, and Yupeng Ge, 212 m asl, *FFAAS1055* (collection no. MY0476).

Diagnosis: Pileus deep gray when young, light drab to drab with age, basidiospores ellipsoid to narrowly ellipsoid, cheilocystidia with finger-like branches at apex. Differs from *M. cristinae* by pruinose pileus, obviously decurrent lamellae, and being weakly intervenose.

Description: *Pileus* 4.2–12.0 mm diam., hemispherical when young, oblate hemispherical to convex with age, umbilicate at the center, margin slightly wavy, revolute, sometimes cracked with age; Castor Gray (LII35’’’’’i) to Pale Violet-Gray (LII59’’’’’d) at the center, gradually paler towards margin to *Pearl Gray (LII35’’’’’f), Smoke Gray (XLVI21’’’’d) when young, Light Drab (XLVI17’’’’b) to Hair Brown (XLVI17’’’’i) at the center, gradually paler towards margin to Pale Smoke Gray (XLVI21’’’’f), Pale Drab-Gray (XLVI17’’’’f) with age; densely covered with pruina when young, sparsely when old, striate towards the center up to 0.5–0.8 R, Castor Gray (LII35’’’’’i) when young, Hathi Gray (LII35’’’’’b), Hair Brown (XLVI17’’’’i) when matured, dry. *Context* White (LIII), fragile, thin. *Lamellae* subdecurrent to decurrent, 0.3–0.9 mm wide, *L* = 12–14, *l* = 1–3, White (LIII) to pale, edge concolorous with face, sometimes non-marginate, weak and irregularly intervened, up to 1/3–1/2 lamellae wide. *Stipe* 10–13 × 0.8–1.0 mm, central, clavate to cylindrical, hollow, fragile, *French Gray (LII45’’’’’f), *Pearl Gray (LII35’’’’’f) in the upper part, darker towards the base to *Cinereous (LII45’’’’’d), Hathi Gray (LII35’’’’’b), finely White (LIII) pruinose and pubescent, base slightly swollen, covered with masses of short and White (LIII) tomentum when young, rarely observed when old. *Odor* and *taste* indistinctive.

*Basidiospores* [108/4/2] 6.3–***7.3***–8.8 × 3.8–4.5–5.7 μm, [Q = 1.43–1.76, Q_m_ = 1.61 ± 0.08] [holotype (40/2/1) 6.4–7.5–8.8 × 3.8–4.7–5.7 μm, Q = 1.43–1.76, Q_m_ = 1.60 ± 0.08], ellipsoid to narrowly ellipsoid, hyaline, smooth (under oil), thin-walled, amyloid. *Basidia* 18–24 × 4–8 μm, two- and four-spored, clavate, hyaline, sterigmata 3.2–5.0 μm in length. *Cheilocystidia* 10–32 × 4–18 μm, clavate, cylindrical, apex with several to fairly numerous finger-like branches, 2.3–22.1 × 0.8–2.1 μm, branches usually with furcate excrescences at apex, 2.3–8.9 × 0.8–1.9 μm, hyaline, thin-walled. *Pleurocystidia* absent. *Pileipellis* a cutis composed of parallel hyphae, 1.9–4.6 μm diam., hyaline, thin-walled, covered with cylindrical excrescences, 1.8–17.9 (29.0) × 0.9–2.3 μm; *pileocystidia* absent. *Lamellae trama* subregular, hyphae 7–19 μm diam., hyaline, dextrinoid. *Stipitipellis* a cutis composed of a hypha, 2.2–4.6 μm diam., hyaline, thin-walled, covered with small cylindrical excrescences, 1.1–6.3 × 0.9–2.1 μm; *caulocystidia* absent. *Clamps* present in all tissues, but rarely observed in context.

Habit and habitat: Scattered on rotten branches in mixed broadleaf–conifer forests, mainly under trees of *Liriodendron*, *Pseudolarix*, and *Pinus*.

Known distribution: Zhejiang Province, China.

Additional material examined: China. Zhejiang Province, Lishui City, 1 August 2021, leg. Renxiu Wei, Binrong Ke, Zhiheng Zeng, Qin Na, and Yupeng Ge, 231 m asl, *FFAAS1054* (collection no. MY0447).

Notes: *M. cristinae* closely resembles *M. digitifurcata* in pileus and stipe color, but it is distinguished by its smooth pileus, adnate lamellae, and markedly intervenose lamellae [26]. *M. pasvikensis* Aronsen, sharing a similar pileus color with *M. digitifurcata*, is differentiated by a densely fibril-covered stipe base, serrulate lamellae, and gelatinized pileipellis and stipitipellis [28]. Similar cheilocystidia shapes are observed in *M. pseudopicta* (J.E. Lange) Kühner and *M. cinerella* (P. Karst.) P. Karst., but *M. pseudopicta* is differentiated from *M. digitifurcata* by its gelatinized pileipellis and stipitipellis, and by possessing larger basidiospores [23,28]; *M. cinerella* is notable for its gelatinized pileipellis and the lighter color of the pileus [28,29]. Microscopic observation reveals a variation in the branch lengths of the cheilocystidia. In specimen *FFAAS1054*, most cheilocystidia display short (0.9–1.9 μm) branched excrescences at the apex, whereas in *FFAAS1055*, cheilocystidia with significantly longer (7.5–22.2 μm) branched excrescences at the apex are typically observed, indicating the various forms of cheilocystidia.

**Figure 15:**
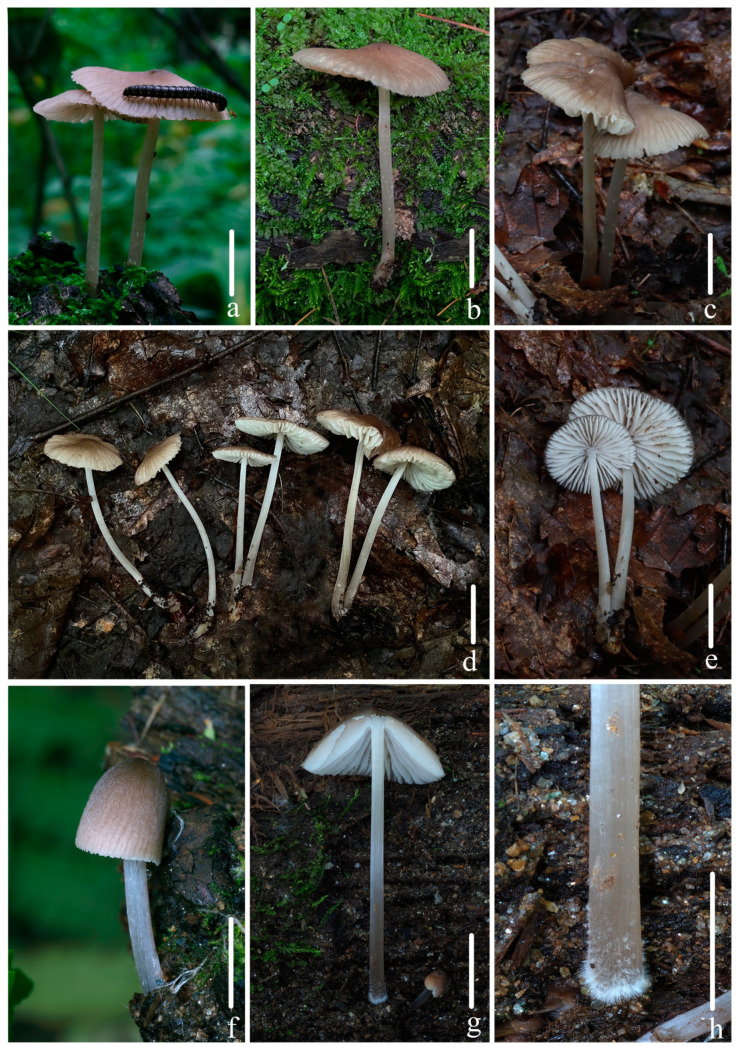
Basidiomata of *Mycena limitis* sp. nov. (**a**,**b**) *FFAAS1056*; (**c**–**e**) *FFAAS1058* (holotype); (**f**–**h**) *FFAAS1057*; (**e**) Lamellae; (**g**) lamellae and surface of stipe are glabrous; (**h**) fibrils in base of stipe. Bars: (**a**–**e**,**g**) = 5 mm; (**f**,**h**) = 10 mm. Photos (**a**–**e**) by Qin Na; (**f**–**h**) Yupeng Ge.

**Figure 16:**
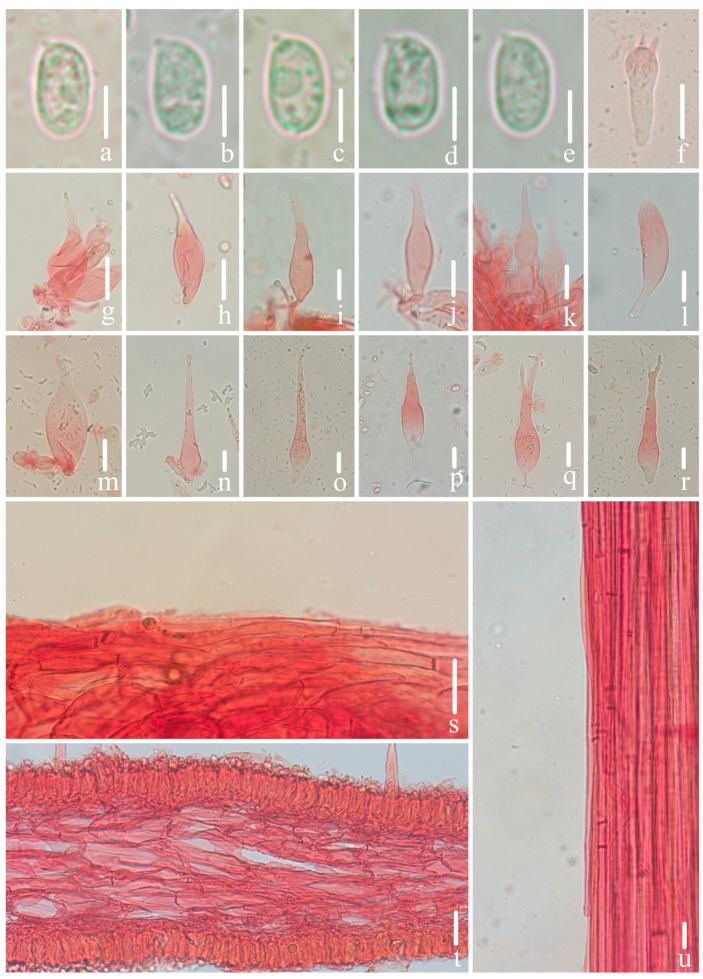
Microscopical features of *Mycena limitis* (*FFAAS1058*, holotype). (**a**–**e**) Basidiospores; (**f**) basidium; (**g**–**l**) cheilocystidia; (**m**–**r**) pleurocystidia; (**s**) pileipellis; (**t**) hymenia and lamellar trama; (**u**) stipitipellis. Bars: (**a**–**e**) = 5 μm; (**f**) = 10 μm; (**g**–**u**) = 20 μm. Structures (**a**–**e**) were rehydrated in 5% KOH aqueous solution, and (**f**–**u**) were stained in 1% Congo red aqueous solution.

**Figure 17:**
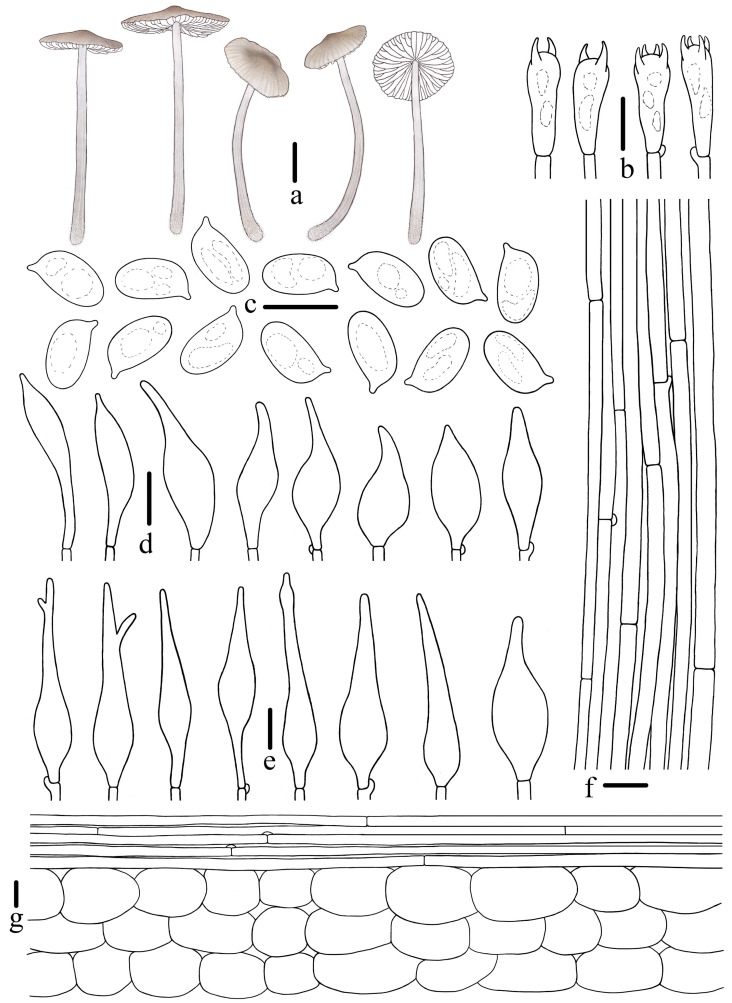
Morphological features of *Mycena limitis* (*FFAAS1058*, holotype). (**a**) Basidiomata; (**b**) basidia; (**c**) basidiospores; (**d**) cheilocystidia; (**e**) pleurocystidia; (**f**) stipitipellis; (**g**) pileipellis and context. Bars: (**a**) = 20 mm; (**b**,**d**–**g**) = 20 μm; (**c**) = 10 μm. Drawing by Renxiu Wei.

***Mycena limitis*** R.X. Wei, L. Zou, Y.P. Ge & Q. Na, sp. nov., Figure 15, Figure 16 and Figure 17.

MycoBank no: 853786

Etymology: The epithet *limitis* is derived from the Latin word *limes*, which emphasizes the morphological similarities with related species and highlights the limited distinguishing features that set this species apart from its close relatives. Additionally, this name pays tribute to the companionship and support provided by a forest ranger, affectionately called brother on the border.

Holotype: CHINA. Heilongjiang Province, Taipinggou National Nature Reserve, Hegang City, 3 July 2023, leg. Renxiu Wei, Li Zou, Menghui Han, Nannan Geng, Tingting Sun, Xinyu Tong, Yawei Li, Zengcai Liu, and Yupeng Ge, 621 m asl, *FFAAS1058* (collection no. GN1786).

Diagnosis: Pileus hair brown when young, olive brown when old, stipe with faint longitudinal stripes, cheilocystidia and pleurocystidia mainly fusiform. Differs from *M. niveipes* by cheilocystidia with tapered apex and stipitipellis with smooth hyphae.

Description: *Pileus* 10–50 mm diam., parabolic and hemispherical when young, oblate hemispherical to plano-convex with age, slightly convex at center, margin slightly serrulate, sometimes cracked at mature; *Hair Brown (XLVI17’’’’i) at center, gradually paler towards margin to Light Drab (XLVI17’’’’i), Pale Smoke Gray (XLVI21’’’’f) when young, Deep Olive (XL21’’’k) at center, gradually paler towards margin to Citrine Drab (XL21’’’i), Pale Olive Buff (XL21’’’f) when matured; covered with finely White (LIII) pubescence when young, then glabrescent, transparent sulcate towards the center up to 0.5–0.6 R, dry. *Context* White (LIII), fragile, thin. *Lamellae* adnate to slightly sinuate, 2.1–5.0 mm wide, *L* = 22–25, *l* = 1–3, White (LIII), edge concolorous with face, inconspicuous intervenose. *Stipe* 59–85 × 2.0–4.1 mm, central, cylindrical, White (LIII) in the upper part, Pale Smoke Gray (XLVI21’’’’f) in the middle part, *Smoke Gray (XLVI21’’’’d), *Olive-Gray (LI23’’’’’b) towards base, hollow, fragile, covered with finely White (LIII) pruina when young, almost glabrous when old, with faint longitudinal stripes, base covered with masses of short and White (LIII) fibrils. *Odor* and *taste* indistinctive.

*Basidiospores* [108/4/3] (8.6) 8.9–10.4–12.6 × 4.7–5.7–6.9 (7.2) μm [Q = 1.65–1.99 (2.01), Q_m_ = 1.82 ± 0.09] [holotype (50/2/1) 8.9–10.7–12.6 × 4.7–***5.8***–6.9 (7.2) μm, Q = (1.65) 1.66–1.99 (2.01), Q_m_ = 1.83 ± 0.09], narrowly ellipsoid, hyaline, smooth (under oil), thin-walled, amyloid. *Basidia* 29–37 × 8–10 μm, clavate, hyaline, two- and four-spored, sterigmata 4.1–6.0 μm in length. *Cheilocystidia* 43–70 × 10–21 μm, fusiform, subfusiform, lanceolate, tapering at apex, base constricted to cylindrical, hyaline, thin-walled, smooth. *Pleurocystidia* 54–123 × 14–26 μm, similar to cheilocystidia, hyaline, thin-walled, occasionally forked in the upper part. *Pileipellis* a cutis composed of parallel hyphae, 3.2–7.0 μm diam., hyaline, thin-walled, smooth; *pileocystidia* absent. *Lamellae trama* regular, hyaline, parallel, hyphae, 6–18 μm diam., dextrinoid. *Stipitipellis* a cutis composed of a hypha, 3.2–5.0 μm diam., hyaline, thin-walled, smooth; *caulocystidia* absent. *Clamps* present in all tissues, but rarely observed in context.

Habit and habitat: Scattered on humus layer and rotten branches in deciduous broad-leaved forests, mainly under trees of *Betula*, *Quercus*, *Styphnolobium*, and *Tilia*.

Known distribution: Heilongjiang Province, Jilin Province, China.

Additional material examined: China. Jilin Province, Changbaishan National Nature Reserve, Yanbian Korean Autonomous Prefecture, leg. Renxiu Wei, Binrong Ke, Chi Yang, Qin Na, and Yupeng Ge, 1 July 2021, 708 m asl, FFAAS1057 (collection no. MY0305); 3 July 2021, 749 m asl, FFAAS1056 (collection no. MY0341).

Notes: *M. niveipes* is similar to *M. limitis* in pileus color and pileipellis with smooth hyphae, but differs by rounded-apex cheilocystidia and the presence of swollen terminal cells in the stipitipellis [23,28]. *M. abramsii* and *M. subcana* A.H. Sm also share a comparable pileus color and cheilocystidia shape but are distinguished from *M. limitis* by the pileipellis and stipitipellis, which are remarkedly covered with excrescences [21,23,28,29]. Similarly, *M. galericulata* exhibits a pileus color similar to *M. limitis*, but it is differentiated by the cheilocystidia and both the hyphae of the pileipellis and stipitipellis featuring excrescences [21,23,28,29]. *M. laevigata* Gillet, while having comparable cheilocystidia, is distinct from *M. limitis* due to its pale grayish-white pileus, the absence of pleurocystidia, and a gelatinized pileipellis [28,29].

## 4. Discussion

Determining the identity of various brownish *Mycena* species based on basidiomata color imposes certain limitations, which consequently may lead to an underestimation of species diversity [11,12,13,14,21,23,28]. Specifically, *M. campanulatihemisphaerica*, *M. limitis*, and *M. kunyuensis* demonstrate close affiliations with *M. abramsii*, *M. algeriensis* Maire, *M. galericulata*, and *M. maculate* P. Karst., all of which are endemic to China [21]. Notably, *M. campanulatihemisphaerica*, *M. kunyuensis*, and *M. abramsii* exhibit a similar basidiomata coloration, with *M. kunyuensis* frequently misidentified as *M. abramsii* due to the analogous coloration of the pileus before morphological anatomy [21,23,28]. Similarly, *M. limitis* is indistinguishable from *M. algeriensis*, *M. galericulata*, and *M. maculata* when identification is based solely on the color of the basidiomata [21,28]. Moreover, species within the complex exhibit a comparable pileus coloration and are subject to variations due to changes in growth period and environment conditions, further complicating the accurate identification of these brownish *Mycena* species [11,12,13,14,69,70,71,72,73,74,75,76,77]. For example, the *M. filopes* complex, as delineated by Arnolds (2015) and Aronsen (2016), is a case in point. [28,78]. A parallel example is found in the *M. pura* complex. While basidiomata coloration has historically served as a criterion for subsection classification within the *M. pura* complex, recent insights by Liu (2023) suggest that the characteristics of the cheilocystidia and pleurocystidia, as well as the presence or absence of pleurocystidia, are critical for species differentiation [11,12,14,79]. Consequently, morphological anatomy is an effective method for the identification of species within the brownish *Mycena* group.

Pileipellis types and cheilocystidia characteristics are integral to the delimitation of brownish *Mycena* species. In the taxonomic framework proposed by Smith (1947) and Maas Geesteranus (1992a, 1992b), pileipellis types and cheilocystidia characteristics are pivotal for sectional categorization [11,12,20]. These characteristics are also crucial criteria for distinguishing various species of brownish *Mycena*, a delineation corroborated by molecular systematics. The phylogenetic tree constructed in this study is divided into 13 clades, and while some clades have lower support rates, the conclusions drawn from the phylogenetic tree remain consistent with those from the morphological anatomy analyses. Notably, *M. campanulatihemisphaerica*, *M. limitis*, and *M. kunyuensis* exhibit smooth cheilocystidia, and cluster phylogenetically into a clade and align closely with the species of sect. *Fragilipedes*, which also exhibit smooth cheilocystidia [17,28]. Moreover, phylogenetic analyses reveal a distinct clade comprising *M. digitifurcata*, which aligns closely with the species of sect. *Rubromarginatae* [26,57]. Sect. *Rubromarginatae* is characterized by marginta lamellae, ranging from deep yellow to dark greenish [11,12]. If the sectional division relies solely on this feature, *M. digitifurcata* would ostensibly not qualify for inclusion within the sect. *Rubromarginatae* due to its lamellae not being marginated [11,12]. However, its pileipellis types and cheilocystidia characteristics are congruent with those of the sect. *Rubromarginatae*, suggesting the need for a broader consideration of morphological characters. Accordingly, *M. digitifurcata* is classified within the sect. *Rubromarginatae*. This classification, proposed by Jadson (2021), identifies *M. cristinae* as a non-marginate lamellae member of the sect. *Rubromarginatae* [26]. Our research aligns with Maas Geesteranus’ (1992a, 1992b) interpretation of the sect. *Rubromarginatae*, which is further supported by the perspectives advanced by Na (2019) [11,12,21]. Specifically, it emphasizes that the delimitation of sections exhibiting colored lamellae edges should predominantly consider variations in pileipellis types and cheilocystidia characteristics. Furthermore, pileipellis and cheilocystidia are present in almost all brownish *Mycena*, reinforcing their utility as key diagnostic features [23,28]. For example, *M. galericulata* can be distinguished from its morphologic relative, *M. algeriensis*, by its tuberculated cheilocystidia, and *M. leptocephala* is distinguished from its close relative *M. polygramma* (Bull.) Gray by smooth pileipellis [11,12,21,23,28]. Consequently, we reaffirm the role of pileipellis types and cheilocystidia characteristics as primary taxonomic criteria within the brownish *Mycena* group, corroborating the classifications of Smith (1947) and Maas Geesteranus (1992a, 1992b) and substantiated by the molecular systematics of Na (2019) [11,12,20,21].

Additionally, this study suggests that *M. oryzifluens* may represent a prospective novel section. Phylogenetically, *M. oryzifluens* is closely related to three established sections, namely: sect. *Exornatae*, sect. *Cyanocephalae*, and sect. *Amictae*. Despite these affiliations, *M. oryzifluens* exhibits distinct morphological characteristics that preclude its classification within these existing sections. These distinguishing features include a dry pileus, the absence of a blue disc at the stipe base, branched cheilocystidia apex, and non-gelatinized pileipellis [11,12,21,28,57]. Consequently, *M. oryzifluens* does not align with any current sectional definitions and suggests the potential for defining a new section. However, due to the lack of enough specimen samples at present, it is prudent not to propose it as a new section within this publication. Future research efforts will collect and examine additional specimens to substantiate the distinctiveness of this taxonomic group.

At present, there are 24 brownish *Mycena* species in China. This study introduces 5 new species, increasing the total to 29. Our analysis of morphological characteristics has identified the types of pileipellis and cheilocystidia as critical distinguishing features for brownish *Mycena*. To facilitate future research and better species distinction, we provide an identification key for the brownish *Mycena* species in China.

**Key to the known species of brownish *Mycena* from China**
1. Basidiospores inamyloid21. Basidiospores amyloid32. Basidiospore broadly ellipsoid, Q = 1.1–1.5
*Mycena hiemalis*
2. Basidiospore narrowly ellipsoid, Q = 1.7–1.9
*Mycena speirea*
3. Pileipellis gelatinized43. Pileipellis not gelatinized104. Stipitipellis hyphae ornamented54. Stipitipellis hyphae smooth65. Pleurocystidia present
*Mycena clavicularis*
5. Pleurocystidia absent
*Mycena polygramma*
6. Caulocystidia absent
*Mycena semivestipes*
6. Caulocystidia present77. Cheilocystidia densely covered with tuberculate excrescences
*Mycena pluteoides*
7. Cheilocystidia smooth88. Pileus with conical spines, basal disc of the stipe present, caulocystidia with outgrowths
*Mycena stylobates*
8. Pileus without any spines, stipe base without disc, caulocystidia without outgrowths99. Pileus convex, depressed at center, lamellae stained with yellow-brown to orange-brown spots
*Mycena subpiligera*
9. Pileus conical to campanulate, umbonate at center, lamellae without any spots
*Mycena amicta*
10. Pileipellis hyphae smooth1110. Pileipellis hyphae ornamented1211. Caulocystidia present
*Mycena algeriensis*
11. Caulocystidia absent
*Mycena limitis*
12. Lamellae flesh pink, pleurocystidia with flesh-pink contents
*Mycena entolomoides*
12. Lamellae white to grayish white, pleurocystidia hyaline1313. Caulocystidia present1413. Caulocystidia absent1814. Cheilocystidia present1514. Cheilocystidia absent1615. Cheilocystidia fusoid to lageniform, with long tapered necks, smooth
*Mycena subcana*
15. Cheilocystidia clavate to obpyriform, densely covered with cylindrical excrescences
*Mycena mirata*
16. Pileus with brown spots in age, stipitipellis hyphae ornamented
*Mycena zephirus*
16. Pileus without any spots in age, stipitipellis hyphae smooth1717. Basidiospore subglobose to broadly ellipsoid, Q = 1.1–1.4
*Mycena oryzifluens*
17. Basidiospore narrowly ellipsoid to cylindrical, Q = 1.6–2.1
*Mycena leptocephala*
18. Pleurocystidia present1918. Pleurocystidia absent2319. Cheilocystidia apically rounded or narrowed into one to several cylindrical or furcate necks2019. Cheilocystidia covered with cylindrical excrescences2120. Stipe grayish brown, basidiospore broadly ellipsoid to ellipsoid, Q = 1.4–1.7 
*Mycena silvae-nigrae*
20. Stipe dark brown, basidiospore narrowly ellipsoid to cylindrical, Q = 1.7–2.1
*Mycena abramsii*
21. Pileus convex, basidiospore narrower than 4 μm
*Mycena luguensis*
21. Pileus conical to campanulate, basidiospore broader than 4 μm2222. Pileipellis with clavate to subglobose terminal cell
*Mycena filopes*
22. Pileipellis without clavate to subglobose terminal cell
*Mycena metata*
23. Cheilocystidia apex narrowed into a cylindrical or furcate neck, rarely with short apical outgrowths2423. Cheilocystidia densely covered with tuberculate or furcate excrescences, or apex with several branches2524. Basidiospore ellipsoid to narrowly ellipsoid, Q = 1.4–1.7
*Mycena kunyuensis*
24. Basidiospore narrowly ellipsoid to cylindrical, Q = 1.7–2.0
*Mycena campanulatihemisphaerica*
25. Cheilocystidia apex with several branches2625. Cheilocystidia densely covered with tuberculate or furcate excrescences2726. Pileus bell-shaped, brown sulcate present, not depressed at center, stipe brown
*Mycena venus*
26. Pileus oblate hemispherical to convex, brown sulcate absent, depressed at center, stipe gray
*Mycena digitifurcata*
27. Pileus and lamellae with red-brown spots when old, cheilocystidia sparsely covered with excrescences
*Mycena maculata*
27. Pileus and lamellae without any spots when old, cheilocystidia densely covered with excrescences2828. Basidiospore narrowly ellipsoid to cylindrical, Q = 1.9–2.2
*Mycena flos-nivium*
28. Basidiospore broadly ellipsoid to ellipsoid, Q = 1.1–1.7
*Mycena galericulata*



## Figures and Tables

**Table 1:** DNA sequences of *Mycena* used in the phylogenetic analysis in this study.

			GenBank NO.		
Species	Voucher/Strain NO.	Locality	ITS	LSU	Reference
*M. abramsii*	HMJAU43606	China	MH396629	MK629355	Direct sub.
*M. abramsii*	HMJAU43282	China	MH396626	MK629348	Direct sub.
*M. aetites*	CBS 221.47	Netherlands	MH856225	MH867752	[51]
*M. aetites*	CBS 222.47	Netherlands	MH856226	MH867753	[51]
*M. aff. discobasis*	DED 8211 (SFSU)	USA	MH414555	MH385331	[52]
*M. aff. discobasis*	BAP 658 (SFSU)	USA	MH414554	MH385330	[52]
*M. aff. murina*	D1.1c	Italy	KJ093496	—	[53]
*M. albiceps*	RA705-6	USA	MK234177	—	[54]
*M. albiceps*	MGW1504	USA	KY744173	MF797661	Direct sub.
*M. algeriensis*	HMJAU43798	China	MK733295	MK722347	Direct sub.
*M. algeriensis*	HMJAU59791	China	OR468696	—	Direct sub.
*M. amicta*	CBS:257.53	Netherlands	MH857184	MH868722	[51]
*M. atkinsoniana*	S.D. Russell iNaturalist # 8545962	USA	MN906081	—	Direct sub.
*M. atkinsoniana*	S.D. Russell MycoMap # 7612	USA	MN906082	—	Direct sub.
*M. bulliformis*	MF74930	USA	MT636954	—	Direct sub.
*M. bulliformis*	JLF9168	USA	MZ007503	—	Direct sub.
*M. bulliformis*	SFSU:BAP 547	USA	KX513844	KX513848	[55]
***M. campanulatihemisphaerica***	**FFAAS1047 Holotype**	**China**	**PP706092**	**PP704692**	**This study**
***M. campanulatihemisphaerica***	**FFAAS1048**	**China**	**PP706093**	**PP704693**	**This study**
***M. campanulatihemisphaerica***	**FFAAS1049**	**China**	**PP706094**	**PP704694**	**This study**
***M. campanulatihemisphaerica***	**FFAAS1050**	**China**	**PP706095**	**PP704695**	**This study**
*M. chlorophos*	CT15101401	China	MH400938	—	Direct sub.
*M. chlorophos*	CT151014	China	MH400939	—	Direct sub.
*M. cinerella*	O73656	Netherlands	GU234146	—	[56]
*M. cinerella*	H6018219	Hungary	MW540670	—	Direct sub.
*M. citrinomarginata*	SHXG	China	OM228755	OM228763	Direct sub.
*M. citrinomarginata*	S.D. Russell iNaturalist # 17474783	USA	ON416970	—	Direct sub.
*M. clavicularis*	HMJAU43611	China	ON791480	ON791547	Direct sub.
*M. coralliformis*	ACL306	Malaysia	—	KJ206962	[57]
*M. cristinae*	JS347	Brazil	MT921381	MT921384	[26]
***M. digitifurcata***	**FFAAS1054**	**China**	**PP706099**	**PP704699**	**This study**
***M. digitifurcata***	**FFAAS1055 Holotype**	**China**	**PP706100**	**PP704700**	**This study**
***M. limitis***	**FFAAS1056**	**China**	**PP706101**	**PP704701**	**This study**
***M. limitis***	**FFAAS1057**	**China**	**PP706102**	**PP704702**	**This study**
***M. limitis***	**FFAAS1058 Holotype**	**China**	**PP706103**	**PP704703**	**This study**
*M. fulgoris*	ACP1690	Malaysia	MG926694	—	[58]
*M. fulgoris*	XAL A. Cortes-Perez 1690	Mexico	NR_163300	—	[58]
*M. galericulata*	HMJAU43035	China	MW222635	—	Direct sub.
*M. galericulata*	HMJAU43845	China	MW222634	—	Direct sub.
*M. galopus*	461E	Poland	MZ078480	—	[59]
*M. galopus*	69	Norway	MW576941	—	[60]
*M. globulispora*	ACP1765	Mexico	MG926696	—	[58]
*M. globulispora*	ACP1704	Mexico	MG926697	—	[58]
*M. haematopus*	1055	Canada	KJ705181	—	Direct sub.
*M. haematopus*	BIOUG24046-B04	Canada	KT695316	—	[61]
*M. inclinata*	Champ-36	Spain	KX449443	—	[62]
*M. inclinata*	AH56011	Spain	ON113881	—	[63]
*M. interrupta*	HMJAU43791	China	MK733300	—	Direct sub.
*M. interrupta*	HMJAU43849	China	MK733301	—	Direct sub.
*M. jingyinga*	LE-BIN 4556	Russia	OP997504	—	Direct sub.
*M. jingyinga*	OMDL00049	USA	OR572512	—	Direct sub.
***M. kunyuensis***	**FFAAS1045 Holotype**	**China**	**PP706090**	**PP704690**	**This study**
***M. kunyuensis***	**FFAAS1046**	**China**	**PP706091**	**PP704691**	**This study**
*M. laevigata*	HMJAU43187	China	MK733302	—	Direct sub.
*M. laevigata*	HMJAU43618	China	MK733304	—	Direct sub.
*M. laevigata*	HMJAU43604	China	MK733303	MK722354	Direct sub.
*M. maculata*	CBS:235.47	Netherlands	MH856231	—	[51]
*M. maculata*	140 m	Norway	MW576905	—	[60]
*M. maculata*	CBS:239.47	Netherlands	MH856234	MH867763	[51]
*M. megaspora*	H6036833	Hungary	MW540680	—	Direct sub.
*M. megaspora*	5702	Russia	MZ754456	—	Direct sub.
*M. meliigena*	NSK 1014985	Russia	OQ216533	—	Direct sub.
*M. meliigena*	S.D. Russell ONT iNaturalist # 98315803	USA	OP455719	—	Direct sub.
*M. metata*	HAY-F-003966	USA	OR858777	—	Direct sub.
*M. metata*	HAY-F-005148	USA	OR882691	—	Direct sub.
*M. mucoroides*	OF76006	Sweden	NR176117	—	Direct sub.
*M. mucoroides*	AAronsen9-060913	Sweden	KU861561	—	[28]
*M. nebula*	ACP1659	Mexico	MG926685	—	[58]
*M. niveipes*	DA-18015	France	OM368073	—	Direct sub.
*M. overholtsii*	iNAT:11409406	USA	MZ146337	—	Direct sub.
*M. overholtsii*	S.D. Russell iNaturalist # 37566563	USA	ON561746	—	Direct sub.
***M. oryzifluens***	**FFAAS1051 Holotype**	**China**	**PP706096**	**PP704696**	**This study**
***M. oryzifluens***	**FFAAS1052**	**China**	**PP706097**	**PP704697**	**This study**
***M. oryzifluens***	**FFAAS1053**	**China**	**PP706098**	**PP704698**	**This study**
*M. pasvikensis*	AAronsen86-12	Sweden	KU861556	—	[28]
*M. pasvikensis*	AAronsen45-13	Sweden	KU861557	—	[28]
*M. picta*	CAFUNDIS iNaturalist 171114596	USA	OR858681	—	Direct sub.
*M. picta*	TUR194167	Hungary	MW540717	—	Direct sub.
*M. plumbea*	AFTOL-ID 1631	USA	DQ494677	DQ470813	[64]
*M. pluteoides*	HMJAU43771	China	MK733307	MK722357	Direct sub.
*M. polycystidiata*	FFAAS0418	China	ON427732	—	[14]
*M. polycystidiata*	FFAAS0421	China	ON427733	—	[14]
*M. polygramma*	CBS 243.47	Netherlands	MH856238	MH867767	[51]
*M. pura*	IS10/11/2000	Denmark	FN394611	—	[65]
*M. renati*	CBS:358.50	Netherlands	MH856658	MH868174	[51]
*M. romagnesiana*	388a	USA	JF908421	—	Direct sub.
*M. romagnesiana*	388f	USA	JF908422	—	Direct sub.
*M. rubromarginata*	UBC F16259	Canada	EF530939	—	Direct sub.
*M. rubromarginata*	CBS:265.48	Netherlands	MH856335	MH867890	[51]
*M. rubromarginata*	CBS:268.48	Netherlands	MH856338	MH867891	[51]
*M. rufobrunnea*	FFAAS0414	China	ON427728	—	[14]
*M. rufobrunnea*	FFAAS0415	China	ON427729	—	[14]
*M. sanguinolenta*	CBS:367.50	Netherlands	MH856662	—	[51]
*M. sanguinolenta*	KUBOT-KRMK-2020-57	India	MW446185	MW446189	Direct sub.
*M. semivestipes*	HMJAU43825	China	MK733308	MK722358	Direct sub.
*M. semivestipes*	HMJAU43830	China	MK733309	—	Direct sub.
*M. seynesii*	71l	USA	JF908469	—	Direct sub.
*M. seynesii*	71h	USA	JF908470	—	Direct sub.
*M. shengshanensis*	FFAAS0424	China	ON427739	—	[14]
*M. shengshanensis*	FFAAS0425	China	ON427740	—	[14]
*M. silvae-nigrae*	HMJAU43815	China	MK733310	MK722359	Direct sub.
*M. sinar*	ACL092	Malaysia	KF537247	—	[66]
*M. sinar*	ACL135	Malaysia	KF537249	—	[66]
*M. subulata*	FFAAS0419	China	ON427735	—	[14]
*M. subulata*	FFAAS0423	China	ON427737	—	[14]
*M. tenuispinosa*	LE 321750	Russia	MK478466	—	Direct sub.
*M. tintinnabulum*	H6008524	Hungary	MW540664	—	Direct sub.
*M. tintinnabulum*	NSK 1017255	Russia	OR242685	—	Direct sub.
*M. venus*	CT20160127	China	MG324368	—	[30]
*M. venus*	CT20160218	China	MG324369	—	[30]
*M. yuezhuoi*	FFAAS0347	China	MW581493	—	[15]
*M. yuezhuoi*	FFAAS0344	China	MW581490	—	[15]
*M. zephirus*	CBS:270.48	Netherlands	MH856339	MH867892	[51]

Remarks: New generated sequences are emphasized in bold; “—” show missing sequence.

## Data Availability

Data are contained within the article.
